# 3D reconstruction of the bronchial tree of the Gray short‐tailed opossum (*Monodelphis domestica*) in the postnatal period

**DOI:** 10.1111/joa.13928

**Published:** 2023-07-27

**Authors:** Kirsten Ferner, Kristin Mahlow

**Affiliations:** ^1^ Museum für Naturkunde, Leibniz‐Institut für Evolutions‐ und Biodiversitätsforschung Berlin Germany

**Keywords:** airway, lung, marsupial, morphogenesis, staining, μCT

## Abstract

Recent didelphid marsupials resemble the assumed mammalian ancestor and are suitable to inform on the evolution of the mammalian lung. This study uses X‐ray computed tomography (μCT) to three‐dimensionally reconstruct the bronchial tree of the marsupial Gray short‐tailed opossum (*Monodelphis domestica*) in order to reveal the timeline of morphogenesis during the postnatal period. The development of the bronchial tree was examined in 37 animals from embryonic day 13, during the postnatal period (neonate to 57 days) and in adults. The first appearance and the branching of lobar, segmental and sub‐segmental bronchioles in the lungs were documented. Based on the reconstructions, the generation of end‐branching airways, the median and maximum generation and the number of branches were calculated for each pulmonary lobe. At birth, the lung of *M. domestica* has a primitive appearance since it consists of a simple system of branching airways that end in a number of terminal air spaces, lobar bronchioles, and first segmental bronchioles are present. During the postnatal period, the volumes of the lung and bronchial tree steadily increase and development, differentiation, and expansion of the bronchial tree takes place. By 14 days, the fundamental bronchial tree consisting of lobar, segmental, and sub‐segmental bronchioles has been established. A mature bronchial tree, including respiratory bronchioles and alveolar ducts is present by day 35. The asymmetry of the right (predominately four lobes) and the left lung (predominately two lobes), as present in *M. domestica*, can be considered as plesiomorphic for Mammalia. In marsupials, the process of branching morphogenesis, which takes place intrauterine in the placental fetus, is shifted to the postnatal period, but follows similar patterns as described in placentals. Lung maturation in general and the branching morphogenesis in particular seems to be highly conservative within mammalian evolution.

## Graphical abstract

This study uses X‐ray computed tomography (μCT) to three dimensionally reconstruct the bronchial tree of the marsupial Gray short‐tailed opossum (*Monodelphis domestica*) in order to reveal the timeline of branching morphogenesis. During the postnatal period, the volumes of the lung and bronchial tree steadily increase and development, differentiation, and expansion of the bronchial tree takes place. Branching morphogenesis seems to be highly conservative within mammalian evolution.

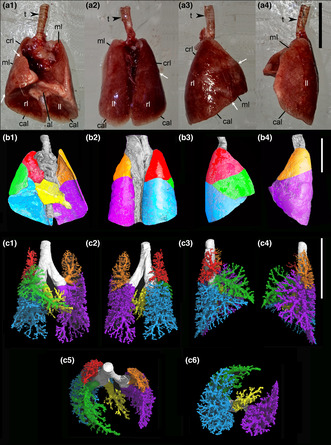

## 1 INTRODUCTION

Survival at birth in mammals depends on the degree of development of the neonate. With the process of birth, the fetus abandons the physical and physiological protection of the uterus and the extra‐embryonic membranes end their functions as the main gaseous exchange system, waste recycle area, and uterine nutrient resource (Ferner et al., 2017). Once born the infant now has to use its own resources to survive risks like hypothermia, desiccation, and depletion of energy reserves (Hughes & Hall, 1988). With birth, the “decision” is made if the organism is capable of surviving, and organ systems (e.g., the cardiorespiratory system, the digestive system, brain) are able to function for extrauterine life (Mess & Ferner, 2010). An essential organ in this context is the lung, because the survivability of the neonate depends to a great extent on the maturity of the respiratory apparatus.

The lung needs to be adequately developed in utero to function as gas exchanging organ. To fulfill the task of gas exchange, the lung has to have a very large surface area possessing a very thin air–blood barrier (Gehr et al., 1978), a mature surfactant system facilitating inflation stability (Clements, 1957; Orgeig et al., 2004; Sano & Kuroki, 2005), an effective vascular system leading the blood to the gas exchange area (Hislop & Reid, 1972), and a tree‐like system of conducting airways ventilating the gas exchange area (Schittny, 2017; Storey & Staub, 1962; Tyler, 1983).

The mammalian lung development can be categorized into five morphological periods (embryonic, pseudoglandular, canalicular, saccular, and alveolar) based on characteristic morphology and has been described in detail elsewhere (Copland & Post, 2004; Schittny, 2017; Tschanz, 2007; Warburton et al., 2010).

This synopsis considers the formation of the bronchial tree during the course of lung development. In the embryonic period, the airways begin their development as a ventral diverticulum budding from the foregut, lined by epithelium of endodermal origin surrounded by mesodermal cells (Lin et al., 2017). In the pseudoglandular period, the preacinar branching pattern of airways and blood vessels is fully established (Jeffery, 1998). Lobar and segmental bronchi are formed; division of segmental airways is progressing fast. During lung branching morphogenesis, three characteristic modes of branching can be distinguished that occur at many different times and positions (Metzger et al., 2008). In humans, the preacinar airway branching pattern is complete by about the 17th week of intrauterine life (Jeffery, 1998). During the following canalicular, saccular, and alveolar periods, the airways continue to increase in size as lung volume increases. The growth of airways continues after birth, resulting in doubling or triplication of airway diameter and length between birth and adulthood (Hislop & Haworth, 1989).

Studies on comparative lung development in various mammalian species showed that the mammalian lung seems to be a highly conservative structure (Ferner et al., 2017; Mess & Ferner, 2010; Nakakuki, 1975, 1980; Szdzuy et al., 2008). The stage of lung development when mammals are born is quite variable, but not the sequence of developmental steps resulting in final lung maturation.

Compared to eutherian neonates, marsupials are generally in earlier stages of development at birth and the primary systems of the marsupial newborn, including digestive, neuronal, immune, and respiratory systems are immature and still under the process of development (Ferner et al., 2017; Renfree, 2006; Szdzuy & Zeller, 2009). Marsupial neonates are similar in development to a late eutherian fetus and the immature lung corresponds to the Carnegie stage 16–17 in the human fetus or E13–E14 in the fetal rat (Fujii et al., 2020; Wang et al., 2009).

Lung developmental studies in marsupial species including the bandicoot (*Isoodon macrourus*; Gemmell, 1986; Gemmell & Little, 1982), Julia creek dunnart (*Sminthopsis douglasi*; Frappell & Mortola, 2000), Fat‐tailed dunnart (*Sminthopsis crassicaudata*; Simpson et al., 2013), Eastern quoll (*Dasyurus viverrinus*; Ferner, 2021a; Hill & Hill, 1955), North American opossum (*Didelphis virginiana*; Krause & Leeson, 1975), brushtail possum (*Trichosurus vulpecula*; Gemmell & Nelson, 1988), quokka (*Setonix brachyurus*; Burri et al., 2003; Makanya et al., 2001, 2003, 2007), tammar wallaby (*Macropus eugenii*; Runciman et al., 1996, 1998), and Gray short‐tailed opossum (*Monodelphis domestica*; Modepalli et al., 2018; Szdzuy et al., 2008) have shown that the lungs of newborns are at the canalicular or saccular period. The lungs comprised of a small number of large air sacs providing insignificant surface area for respiration and are considered as functionally immature (Mess & Ferner, 2010). Many of the structural prerequisites for a functioning lung (e.g., a large surface area for gas exchange), an appropriately developed vasculature, a coordinated neuromuscular effort, and most important a fully developed conductive airway tree, are missing in newborn marsupials, resulting in a neonate with a limited respiratory performance that must rely to varying degrees on transcutaneous gas exchange (Ferner, 2018, 2021b; MacFarlane & Frappell, 2001; Simpson et al., 2011, 2013).

Most of the studies of lung development in marsupial species have placed emphasis on the postnatal development of alveoli. Only few histological and ultrastructural studies provide information about airway structures in marsupials (Cooke & Alley, 2002; Cope et al., 2001; Krause & Leeson, 1973, 1975; Szdzuy, 2006; Tucker, 1974). Information about the development of the bronchial tree originate primarily from eutherian species (Bucher & Reid, 1961; Fujii et al., 2020; Horsfield et al., 1987; Jeffery, 1998; Kitaoka et al., 1996; Metzger et al., 2008; Warburton, 2021).

Traditionally, the bronchial tree of mammals was studied by producing celloidin or silicone rubber casts of the conducting portion of the airways (e.g., Ishaq, 1980; Nakakuki, 1992, 1993a, 1994a; Perry et al., 2000). However, this technique is complicated by the pressure of the lung, which proves to be difficult to assess in small lungs, such as marsupials (Cooke & Alley, 2002). In more recent years, the establishment of μCT techniques in combination with 3D remodeling opened new possibilities to examine and describe the three‐dimensional structure of the mammalian bronchial tree (e.g., Bell et al., 2018; Counter et al., 2013; Fujii et al., 2020; Nagashima et al., 2015, 2017; Onuma et al., 2001; Tawhai et al., 2004). However, a three‐dimensional reconstruction of the bronchial tree in a marsupial species is missing so far. The only study examining the developing lung of a marsupial species by phase‐contrast imaging methods and μCT scans resulted in three‐dimensional volume rendering of the entire lung, demonstrating the comprehensive modifications of the air sacs, but neglecting the bronchial tree (Simpson et al., 2013).

The ancestral condition of a mammalian neonate is interpreted to be similar to the state of organ development found in the newborns of marsupials and monotremes (Ferner et al., 2017). The Gray short‐tailed opossum (*Monodelphis domestica*) resembles both the supposed marsupial and mammalian ancestor (Deakin et al., 2013; Ferner et al., 2017; Szdzuy & Zeller, 2009) and is suitable to inform on the evolution of the mammalian lung.

Therefore, the Gray short‐tailed opossum offers a unique opportunity for a better understanding of the development of the mammalian bronchial tree given the unique finding that, in contrast to eutherian mammals, the canalicular stage of lung development is encountered postnatally and the entire process of postnatal lung development including the formation of the bronchial tree occurs in a ventilated functioning state. Since the adult lung structure of *Monodelphis domestica* and that of eutherian species resemble each other, it can be hypothesized that the development of the bronchial tree in *Monodelphis* domestica follows similar developmental trajectories as described for eutherian species, however decelerated and postponed in the postnatal period.

This study uses X‐ray computed tomography (μCT) to three dimensionally reconstruct the bronchial tree of *Monodelphis domestica* in order to reveal the timeline of morphogenesis during the postnatal period.

## 2 MATERIALS AND METHODS

### 2.1 Specimen collection

The Gray short‐tailed opossums (*Monodelphis domestica*) used for this study were obtained from a laboratory colony established at the Museum für Naturkunde Berlin. After controlled mating, the females were checked for offspring when approaching full‐term (13–14 days). Young ranging from birth to 57 postnatal days and adults were collected, weighed, and killed by anesthetic overdose with isoflurane under animal ethics permit approved by the Animal Experimentation Ethics Committee (registration number: T0202/18). One female was killed short before term by 13 days post coitum (dpc) and the embryos were dissected and fixed by Karnovsky fixative (Mulisch & Welsch, 2015). A total of 37 animals between 13 dpc and adults were studied using X‐ray computed tomography (μCT). The specifics and numbers of the specimens are summarized in Table 1. Additional four animals (5 dpn, 7 dpn, 28 dpn, and adult) were investigated by scanning electron microscopy (SEM). Details for these animals are provided in Szdzuy (2006).

**TABLE 1:** Gray short‐tailed opossum (*Monodelphis domestica*) specimens examined in this study.

Age (days)	Species number	Body weight (g)	Medium	Staining	Lung volume (mm^3^)	Bronchial tree volume (mm^3^)
13 dpc	2095d^a^	—	Araldite	—	0.63	0.03
	2095e	—	Karnovsky	PTA	0.48	0.03
	2095f^a^	—	Araldite	—	0.36	0.03
	2095g	—	Karnovsky	PTA	0.66	0.03
Median (range)					0.56 (0.30)	0.03 (0.00)
Neonate	1965_1^a^	0.12	Araldite	—	2.70	0.21
	2350_1	0.13	Karnovsky	PTA	1.73	0.09
	2350_3	0.13	Karnovsky	PTA	1.98	0.11
	2350_7	0.13	Karnovsky	PTA	2.74	0.24
Median (range)		0.13 (0.01)			2.34 (1.01)	0.16 (0.15)
4 dpn	1995_5	0.22	Bouin/70% Eth.	Iodid	4.10	0.16
	2257_6	0.21	Karnovsky	PTA	4.19	0.23
	2257_8	0.21	Karnovsky	PTA	5.60	0.24
Median (range)		0.21 (0.02)			4.19 (1.50)	0.23 (0.08)
7 dpn	1987_5	0.34	Bouin/70% Eth.	PTA	6.08	0.42
	2383_2	0.28	Karnovsky	PTA	5.63	0.68
	2383_4^a^	0.27	Araldite	—	5.56	0.62
Median (range)		0.28 (0.07)			5.63 (0.52)	0.62 (0.26)
11 dpn	1993_2	0.69	Bouin/70% Eth.	Iodid	20.58	1.00
	1993_4^a^	0.64	Araldite	—	17.58	0.63
	1993_3^a^	0.73	Araldite	—	21.42	1.44
Median (range)		0.69 (0.09)			20.58 (3.84)	1.00 (0.81)
14 dpn	1994_8	0.99	Bouin/70% Eth.	PTA	27.78	1.92
	1994_9	0.98	Bouin/70% Eth.	PTA	24.87	2.10
	1994_10	1.03	Formol/70% Eth.	PTA	24.64	1.76
Median (range)		0.99 (0.05)			24.87 (3.14)	1.92 (0.34)
21 dpn	2040	2.43	Bouin/70% Eth.	PTA	71.14	4.00
	2037^a^	2.34	Araldit	—	58.84	3.90
	2036^a^	2.26	Araldit	—	51.42	3.28
Median (range)		2.34 (0.17)			58.84 (19.72)	3.90 (0.72)
28 dpn	2059	4.22	Bouin/70% Eth.	PTA	189.75	13.08
	2060	4.16	Bouin/70% Eth.	Iodid	208.62	14.25
Median (range)		4.19 (0.06)			199.19 (18.87)	13.67 (1.17)
35 dpn	2065	7.25	Bouin/70% Eth.	Iodid	388.59	28.71
	2405	6.08	Karnovsky	PTA	344.00	23.16
	2194	7.68	Karnovsky	PTA	493.68	21.30
Median (range)		7.25 (1.60)			388.59 (149.68)	23.16 (7.41)
49 dpn	2049	13.64	Bouin/70% Eth.	Iodid	504.03	25.38
	2402	11.59	Karnovsky	PTA	452.66	24.78
	2403	11.09	Karnovsky	PTA	408.96	23.58
Median (range)		11.59 (2.55)			452.66 (95.07)	24.78 (1.80)
57 dpn	2179	31.58	Bouin/70% Eth.	Iodid	1176,68	103.51
	2413	14.81	Karnovsky	PTA	663.60	43.85
	2416	18.53	Karnovsky	PTA	790.89	68.88
Median (range)		18.53 (16.77)			790.89 (513.08)	59.28 (59.67)
Adult	2095	87.22	Karnovsky	PTA	2948.94	213.35
	2117	69.47	Karnovsky	PTA	2307.41	210.86
	2419	66.46	Karnovsky	PTA	2631.63	269.91
Median (range)		69.47 (20.76)			2631.63 (556.28)	213.35 (59.06)

*Note*: Body weights and volumes of the lung and bronchial tree are presented.

Abbreviations: dpc, days post coitum; dpn, days post natum; Eth., Ethanol; PTA, tungstic acid.

^a^ Specimen processed for electron microscopy.

### 2.2 Lung fixation

Young ranging from neonate (defined as the first 24 h at the day of birth) to 28 days post natum (dpn) were decapitated, to allow better lung fixation via the trachea, and the whole animals were immersed in Karnovsky (2 g paraformaldehyde, 25 mL distilled water, 10 mL 25% glutaraldehyde, and 15 mL 0.2 M phosphate buffer) or Bouin's solution (picric acid, formalin, 100% acetic acid, and 15: 5: 1; Mulisch & Welsch, 2015) for a longer period of time and afterward rinsed in 70% ethanol. Lungs of animals from 35 dpn to adults were fixed via the trachea. The animal was placed in a supine position and the trachea was exposed via a midline incision, a cannula with polyethylene catheter tubing of an appropriate outer diameter was introduced into the trachea, and the lungs were then fixed via the trachea with Karnovsky solution at a pressure head of 20 cm, until fixative was emerging from nostrils and mouth.

Some animals were fixed for transmission and scanning electron microscopy (TEM and SEM). In small stages from neonate to 11 dpn, the upper part of the trunk was cut in two halves and in older stages, the lung was dissected.

The specimens for electron microscopy were fixed in 2.5% glutaraldehyde buffered in 0.2 M cacodylate (pH 7.4) for 2 h and rinsed with 0.1 M cacodylate buffer. For TEM, the samples were postfixed in 1% osmium tetroxide and embedded in epoxy resin (Araldite). For SEM, the samples were dried in an ascending alcohol series (30%–100%), “critical‐point‐dried,” mounted on a carrier plate, sputtered with gold–palladium‐particles, and finally viewed in a scanning electron microscopy (LEO 1450 VP, Carl Zeiss NT GmbH).

### 2.3 Preparation for μCT

For visualization of the lungs in X‐ray computed tomography, the specimens had to be stained in advance. Different staining protocols were tested and used at different age stages (Table 1). Staining with tungstic acid (PTA) was either performed in ethanol with 1% PTA for 21–42 days (full body specimens of 14 to 28 dpn) or in an aqueous solution (Karnovsky fixative) starting with 0.5% for 7–20 days and increased afterward to 1% resulting in a staining period up to 30 days (Metscher, 2009). Separated lungs of older stages and adult specimens were stained in 1% Iodine (Gignac et al., 2016) to test staining differences and shrinking effects, which could not be detected. The differences in staining periods and staining concentration depended on the respective specimen size and preparation.

The specimens processed for electron microscopy could be scanned without further processing.

### 2.4 μCT imaging

The prepared specimens were subjected to micro‐tomographic analysis at the Museum für Naturkunde Berlin (lab reference ID SCR_022585) using a Phoenix nanotom X‐ray|s tube (Waygate Technologies, Baker Hughes, Wunstorf, Germany; equipment reference ID SCR_022582) at 70–110 kV and 75–240 μA, generating 1440–2000 projections (Average 3–6) with 750–1000 ms per scan or a YXLON FF85 (equipment reference ID SCR_020917) with transmission beam for bigger specimens at 90–110 kV and 100–150 μA, generating 2000 projections (Average 3) with 250–500 ms. The different kV, μA, and projection settings depended on the respective machine and specimen size, which is also responsible for the range of the effective voxel size between 1.5 and 20.1 μm. The cone beam reconstruction was performed using the datos|x 2 reconstruction software (Waygate Technologies, Baker Hughes; datos|x 2.2) or Nexus reconstruction software, respectively.

### 2.5 3D reconstruction

**FIGURE 1:**
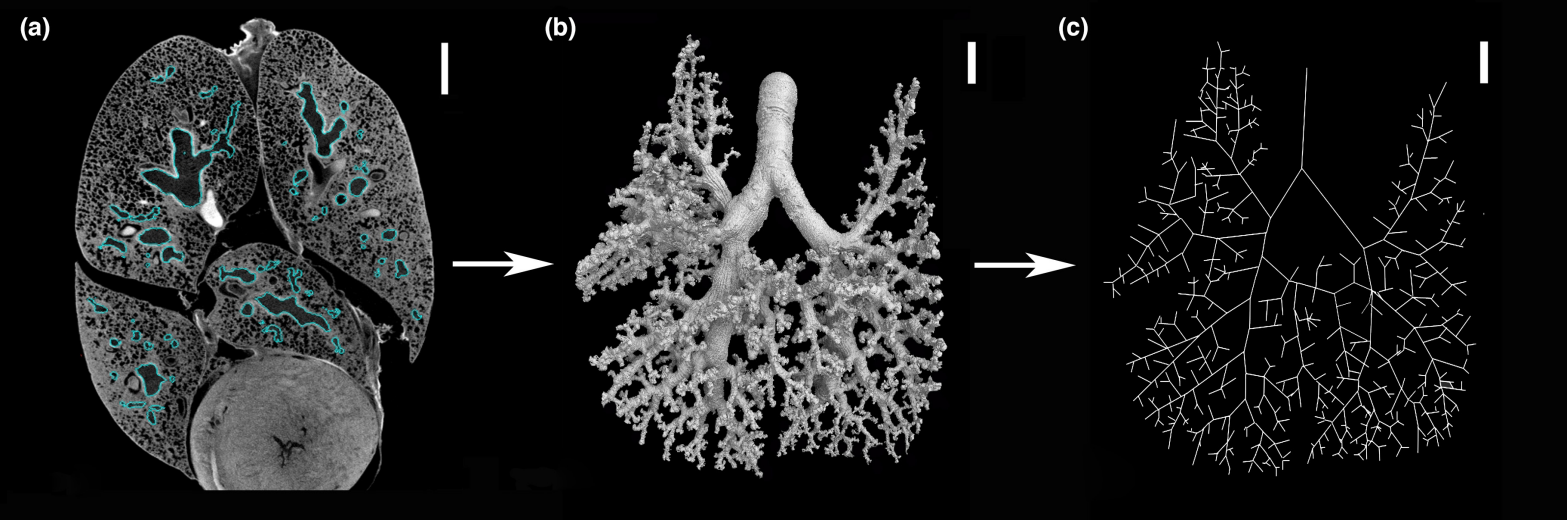
Three‐dimensional reconstruction of the bronchial tree using 3D X‐ray μCT. (a) Representative 3D X‐ray μCT image in the transverse section at 35 dpn (2065). (b) The bronchial tree was reconstructed. (c) Centerline based on reconstruction. Scale bar = 1.5 mm.

The 3D volume processing was done with the software Volume Graphics Studio Max Version 3.5 (Volume Graphics GmbH). 3D X‐ray tomography data were analyzed in detail as serial two‐dimensional (2D) and reconstructed to three‐dimensional (3D) images. In the 2D images, organ tissues appeared in different shades of gray according to the density of the tissue. Formerly air‐filled regions, such as the bronchial tree and air spaces of the lung appeared black. The outline of the lung and the bronchial tree was reconstructed on a slice‐by‐slice basis and regions of interest (ROI) were created. The bronchial tree was reconstructed from the bifurcation of the trachea to the point where the tubular airways opened to the terminal air spaces, which was defined as end of the bronchial tree. In the early postnatal stages, the end‐branches were lobar bronchioles or segmental bronchioles, but in later stages, end‐branches consisted of numerous terminal bronchioles leading to the periphery of the lung. The volumes of the ROIs of the total lung and of the bronchial tree were calculated by the program Volume Graphics and indicated by mm^3^ with an accuracy of two digits after the decimal point. The values are presented in Table 1. The final 3D reconstruction of the bronchial tree was transformed into a centerline model of the airways visually showing the generation of the branches (Figure 1). Based on the centerline model (Figure 9), the median and maximum generation and the number of branches were calculated for each pulmonary lobe in animals from 13 dpc to 57 dpn and the values are presented as median and range (Table S1).

## 3 RESULTS

### 3.1 Morphology of the pulmonary lobes

**FIGURE 2:**
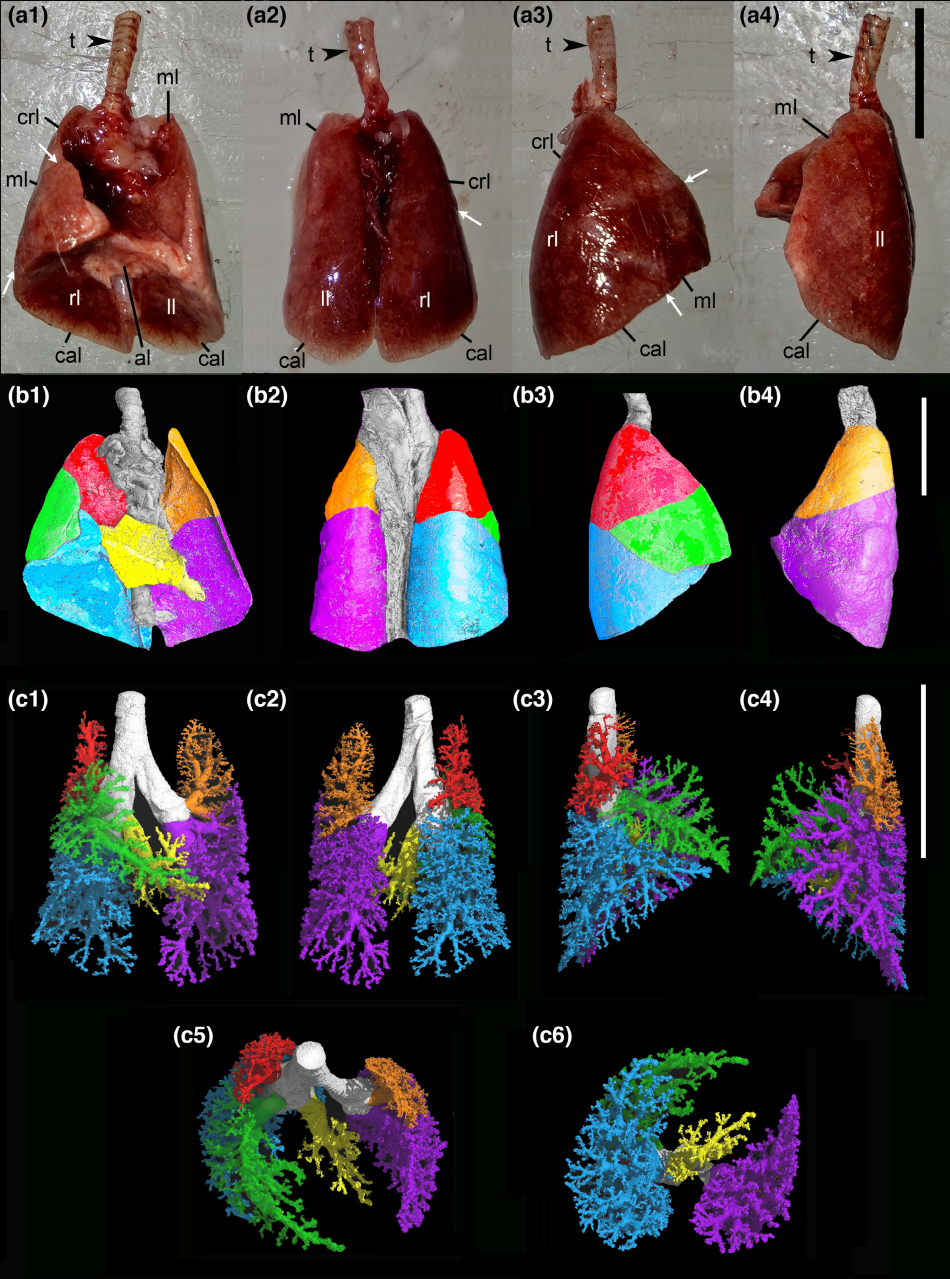
Representation of the lung of *Monodelphis domestica* at 57 dpn. Ventral (A1), dorsal (A2) and lateral views from right (A3) and left side (A4) of the freshly dissected lung and external view of a μCT scan (B1–B4). In (A), the pulmonary lobes and fissures are marked by labeling and white arrows. Reconstruction of the bronchial tree with differentiation of the pulmonary lobes are shown in ventral, dorsal, and lateral views (C1–C4) and additional perspectives from the cranial (C5) and caudal side (C6) are provided. In (B) and (C), the pulmonary lobes are indicated by colors: right lung—cranial lobe (red), middle lobe (green), accessory lobe (yellow), and caudal lobe (blue); left lung—middle lobe (orange) and caudal lobe (purple). al, accessory lobe; cal, caudal lobe; cr, cranial lobe; ll, left lung; ml, middle lobe; rl, right lung; t, trachea. Scale bar = 1 cm.

The lung anatomy of *Monodelphis domestica* is illustrated in Figure 2. Terminology‐identifying lung lobes are consistent with the *Nomina Anatomica Veterinaria* (1994). The right lung consists of a cranial lobe, middle lobe, caudal lobe, and an accessory lobe (Figure 2). All four lobes of the right lung are separated by interlobular fissures. The cranial lobe is pyramidal in shape and the medial surface of this lobe covers the right cranial vena cava and the cranial part of the right atrium of the heart. The middle lobe of the right lung is triangular in shape. The cranial extent of this lobe covers the caudal part of the right atrium and encompasses caudoventrally the right ventricle. The middle lobe is separated from the cranial lobe by a vertical fissure. The caudal lobe of the right lung is triangular in shape and separated from the middle lobe by an oblique fissure. It is approximately twice as large as the middle lobe. The base of the caudal lobe appears smooth and concave, resulting from the base of the lungs lying against the cranial surface of the diaphragm. The accessory lobe and the caudal vena cava are located medial to the caudal lobe. The accessory lobe is shaped like an irregular pyramid. The caudodorsal part of the heart is adjoining the cranial surface of the accessory lobe, resulting in a prominent cardiac impression on this lobe. As in the caudal lobes, also the caudal surface of the accessory lobe is molded to the convex, cranial surface of the diaphragm. Filling the thoracic space efficiently, the right and left surfaces lie adjacent to the medial surfaces of the caudal lobes of the right and left lungs. The left surface of the accessory lobe is elongated and rests within the mediastinal recess.

The left lung consists of middle and caudal lobes (Figure 2). The lobes are not identifiable superficially, since the lobes are not separated from one another by a fissure. However, the representation of the lobar bronchioles shows clearly a subdivision of the lung in middle and caudal lobes (Figure 2c). Medially, the lung covers the left atrium and a portion of the left ventricle of the heart.

### 3.2 Development of the bronchial tree

**FIGURE 3:**
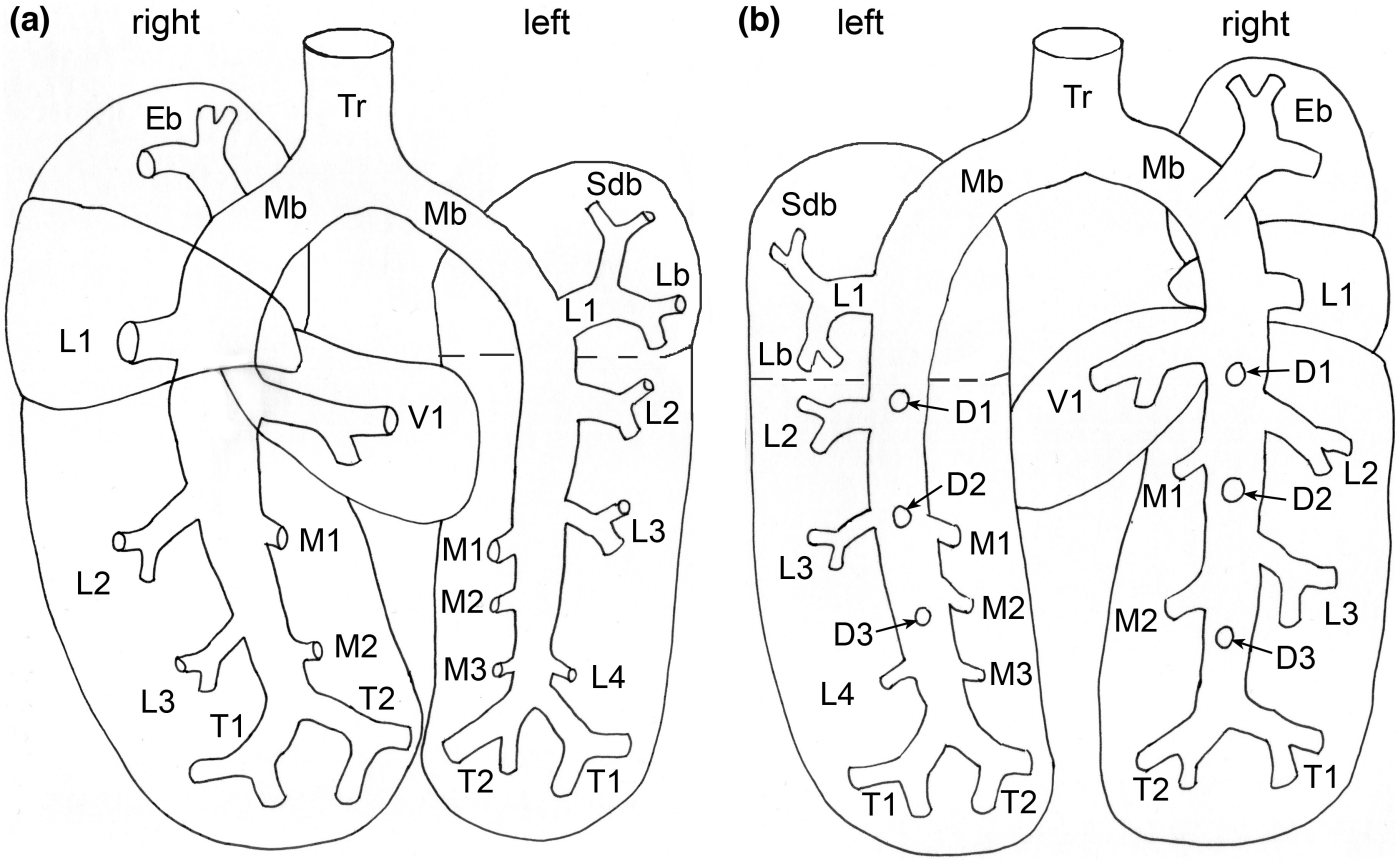
Schema of the bronchial tree of *Monodelphis domestica* in ventral (a) and dorsal (b) view. D1–D3, dorsal row bronchiole; Eb, eparterial bronchiole; L1–L4, lateral row bronchiole; Lb, lingular bronchiole; M1–M3, medial row bronchiole; Mb, main bronchus; Sdb, superior division bronchiole; T1–T2, terminal bronchiole; Tr, trachea; V1, ventral row bronchiole.

**FIGURE 4:**
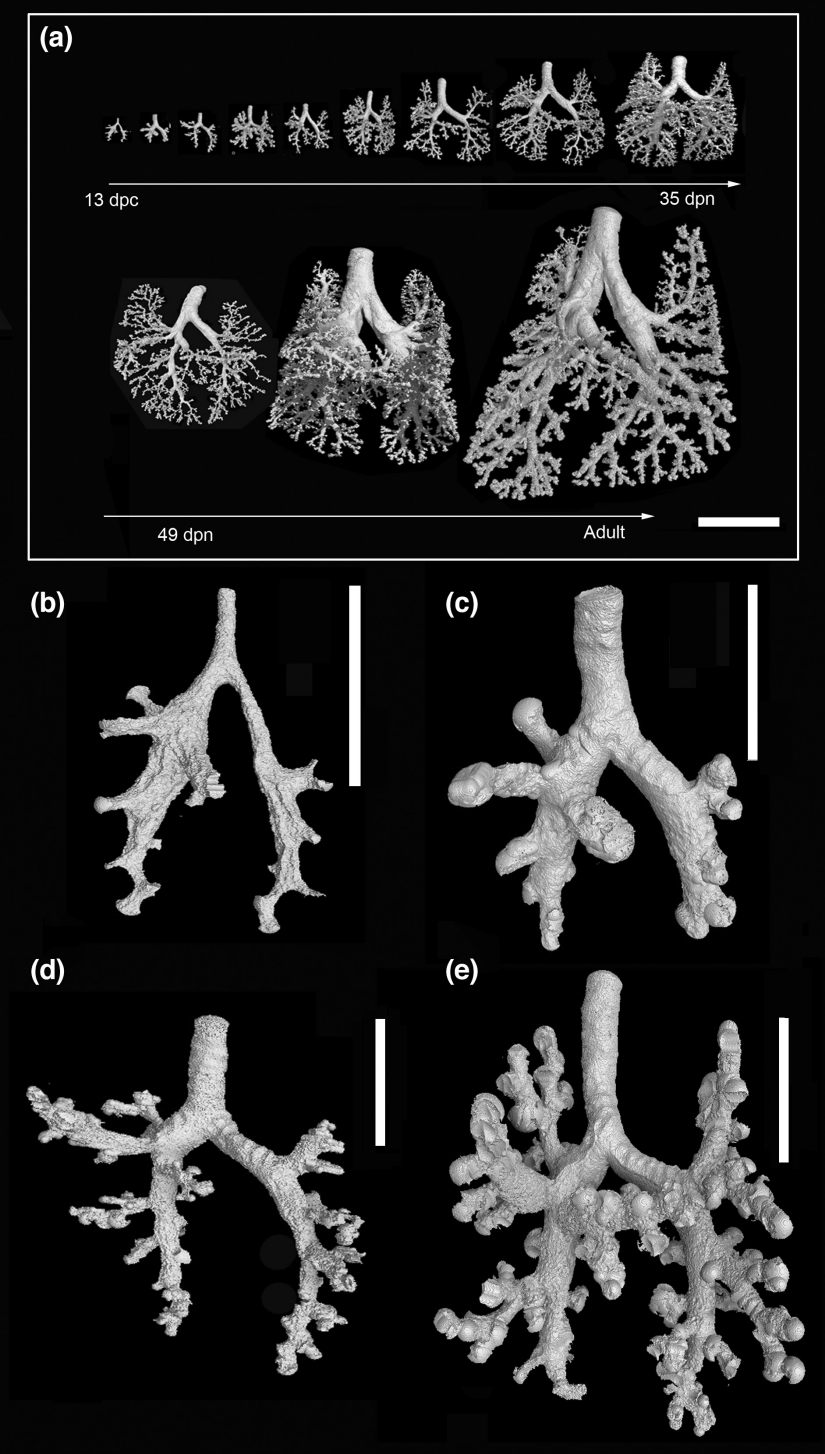
3D reconstructions of the bronchial trees according to growth presented with the same scale highlight the impressive size increase of the developing lung from 13 dpc to adult (a). Representative reconstructions of the bronchial tree of *Monodelphis domestica* at 13 dpc (b), in the neonate (c), at 4 dpn (d), and at 7 dpn (e). The scale bar is 5 mm in (a), 0.5 mm in (b), and 1 mm in (c–e).

**FIGURE 5:**
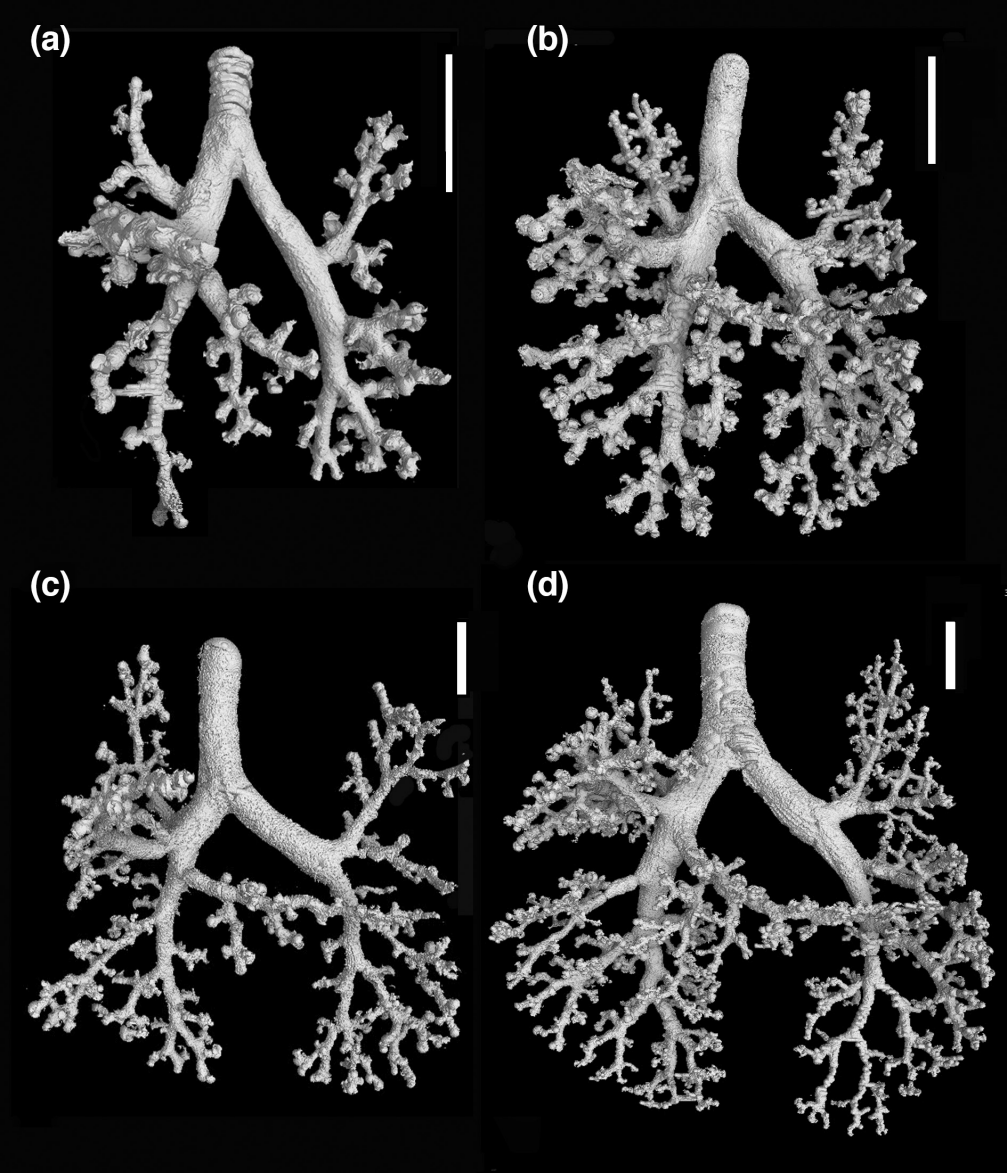
Representative reconstructions of the bronchial tree of *Monodelphis domestica* at 11 dpn (a), at 14 dpn (b), at 21 dpn (c), and at 28 dpn (d). The scale bar is 1 mm.

**FIGURE 6:**
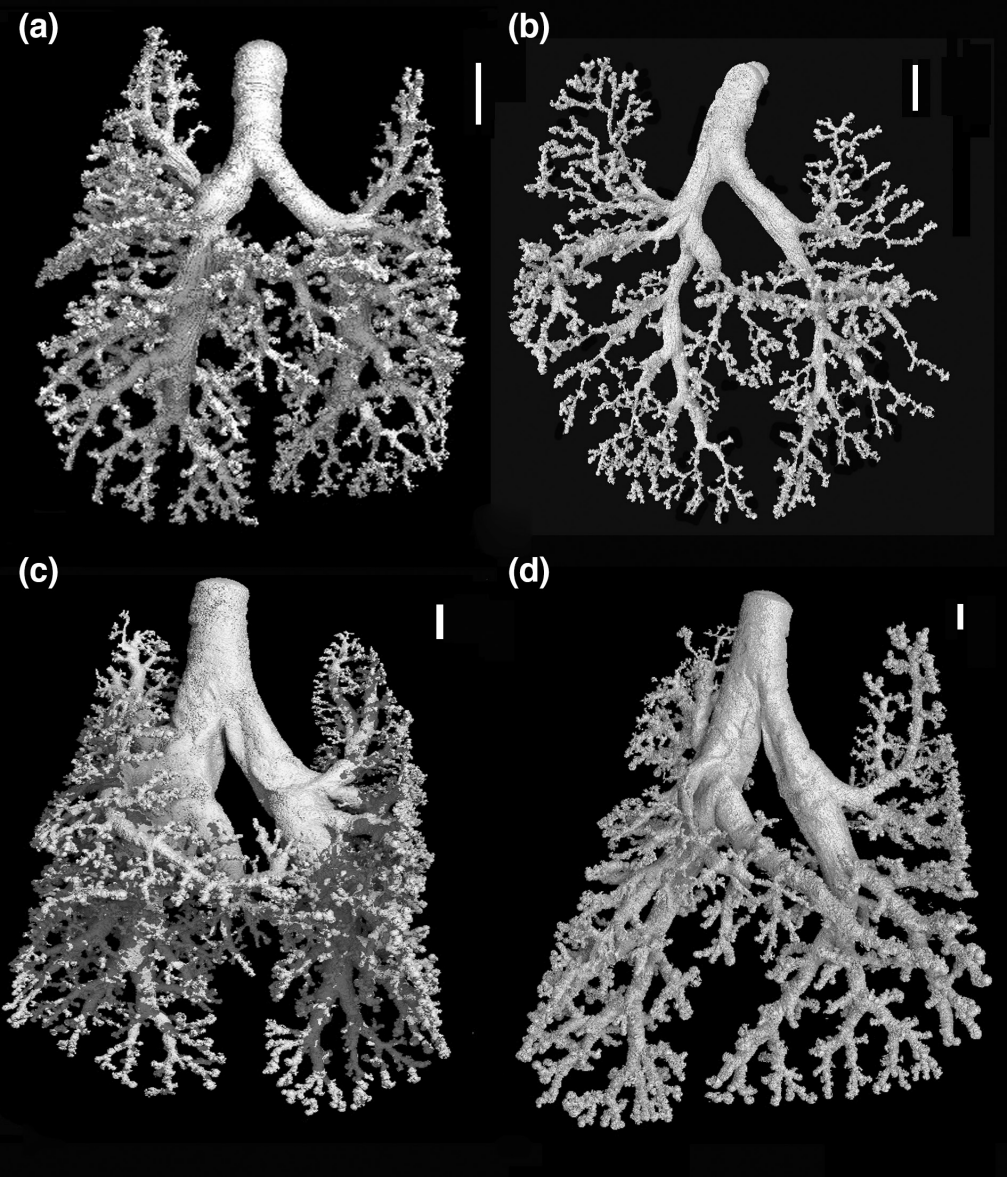
Representative reconstructions of the bronchial tree of *Monodelphis domestica* at 35 dpn (a), at 49 dpn (b), at 57 dpn (c), and in the adult (d). The scale bar is 1 mm.

**FIGURE 7:**
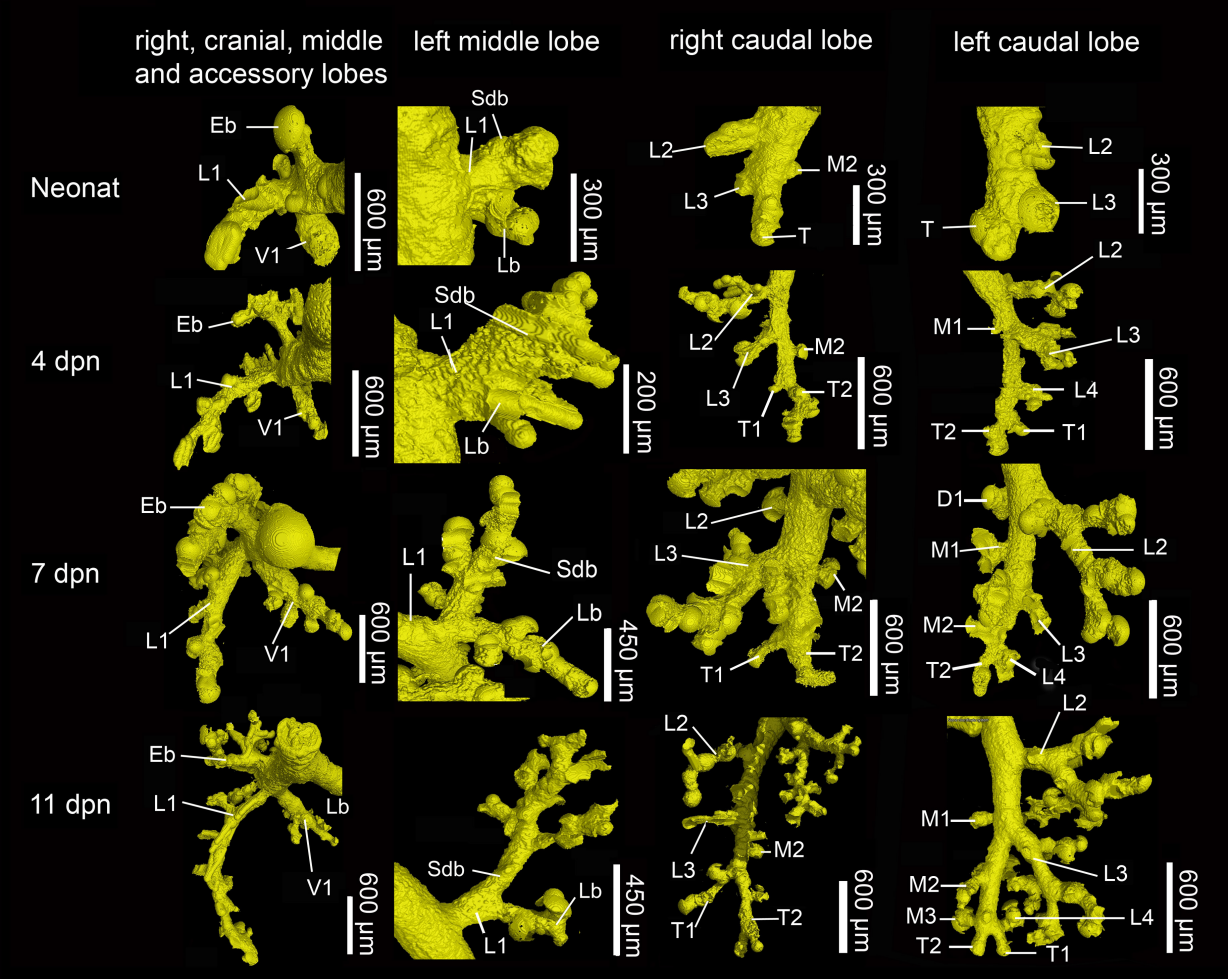
Details of the developing bronchial tree from neonate to 11 dpn. In the neonate *Monodelphis domestica*, a simple bronchial tree consisting of lobar bronchioles in the right cranial, middle, and accessory lobes and the left middle lobe can be seen. Branching of segmental bronchioles takes place at 4 dpn. Starting from 7 dpn, subsegmental bronchioles branch off. Eb, eparterial bronchiole; L1–L4, lateral row bronchiole; Lb, lingular bronchiole; M1–M3, medial row bronchiole; Sdb, superior division bronchiole; T1–T2, terminal bronchiole; V1, ventral row bronchiole.

**FIGURE 8:**
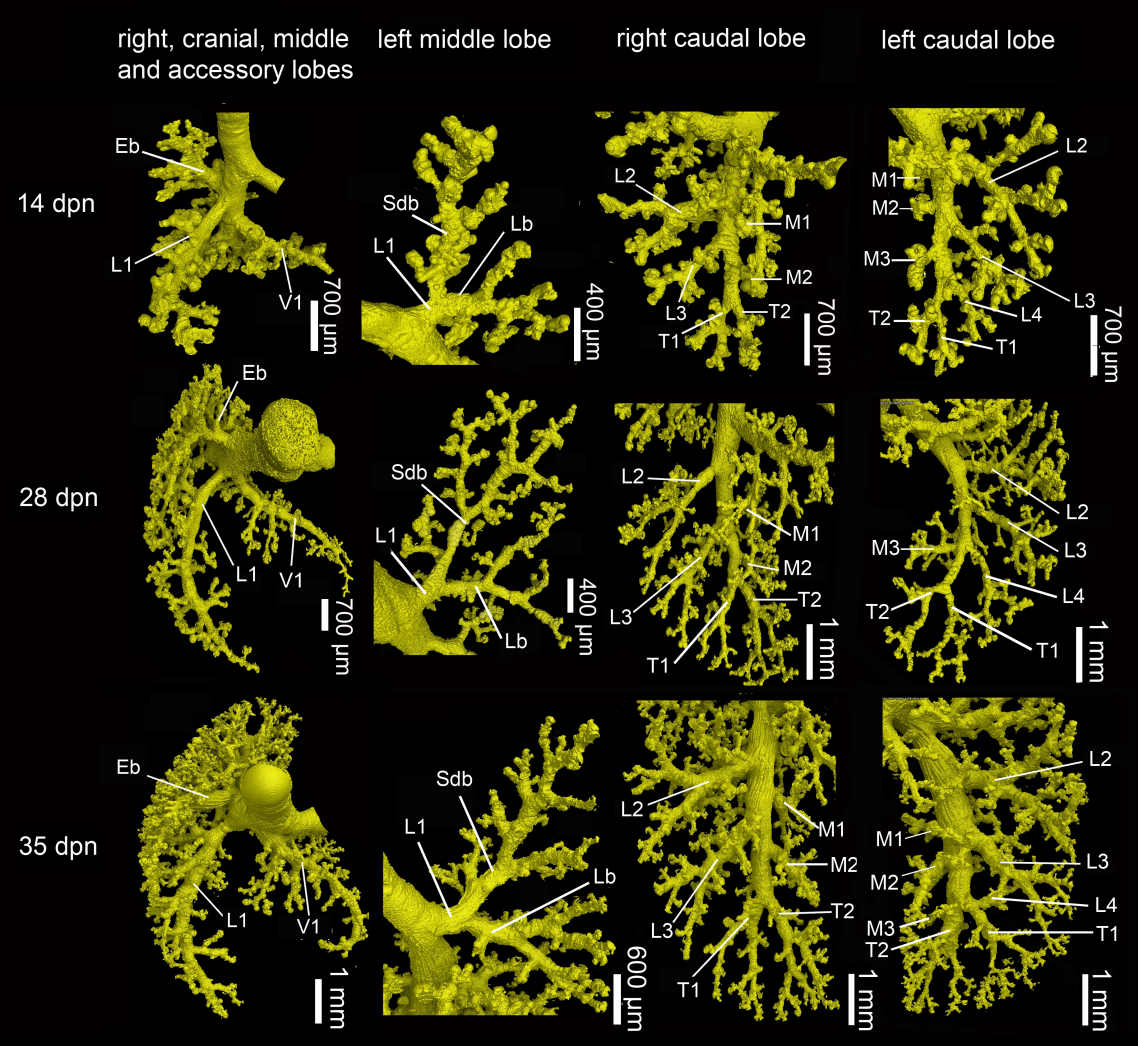
Details of the developing bronchial tree from 14 dpn to 35 dpn. In the 14 dpn old lung, many subsegmental bronchioles exist and the fundamental bronchial tree is formed. By 35 dpn, a structurally mature bronchial tree exists. Eb, eparterial bronchiole; L1–L4, lateral row bronchiole; Lb, lingular bronchiole; M1–M3, medial row bronchiole; Sdb, superior division bronchiole; T1–T2, terminal bronchiole; V1, ventral row bronchiole.

**FIGURE 9:**
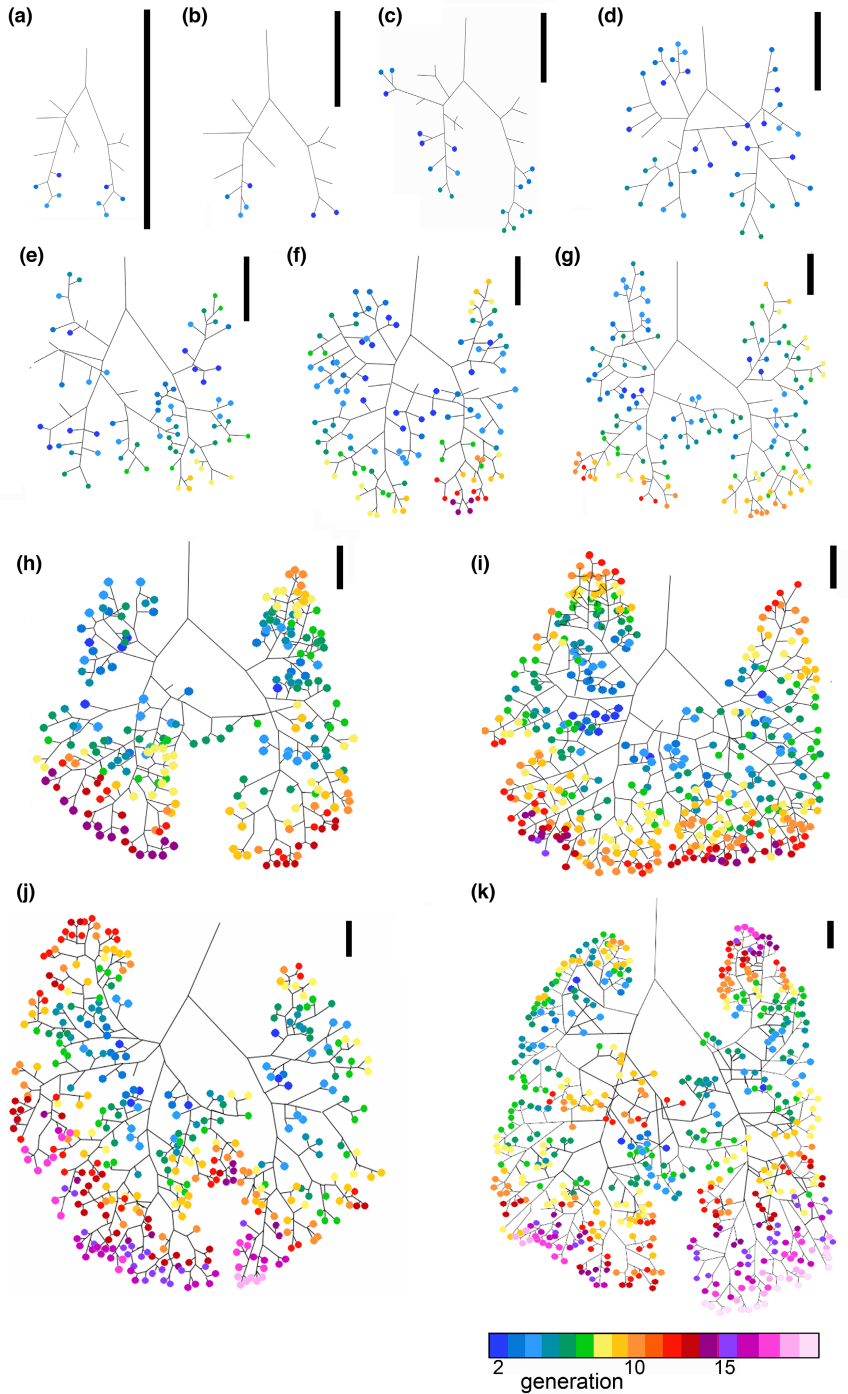
Centerline reconstructions showing generations of end‐branching bronchioles (rainbow color) for 13 dpc (a), neonate (b), 4 dpn (c), 7 dpn (d), 11 dpn (e), 14 dpn (f), 21 dpn (g), 28 dpn (h), 35 dpn (i), 49 dpn (j), and 57 dpn (k). The colored circles indicate generations of each end‐branch when the lobar bronchiole was defined as 0th branch. Color bar indicates the end‐branching generation with the corresponding color. Scale bar = 1 mm.

**FIGURE 10:**
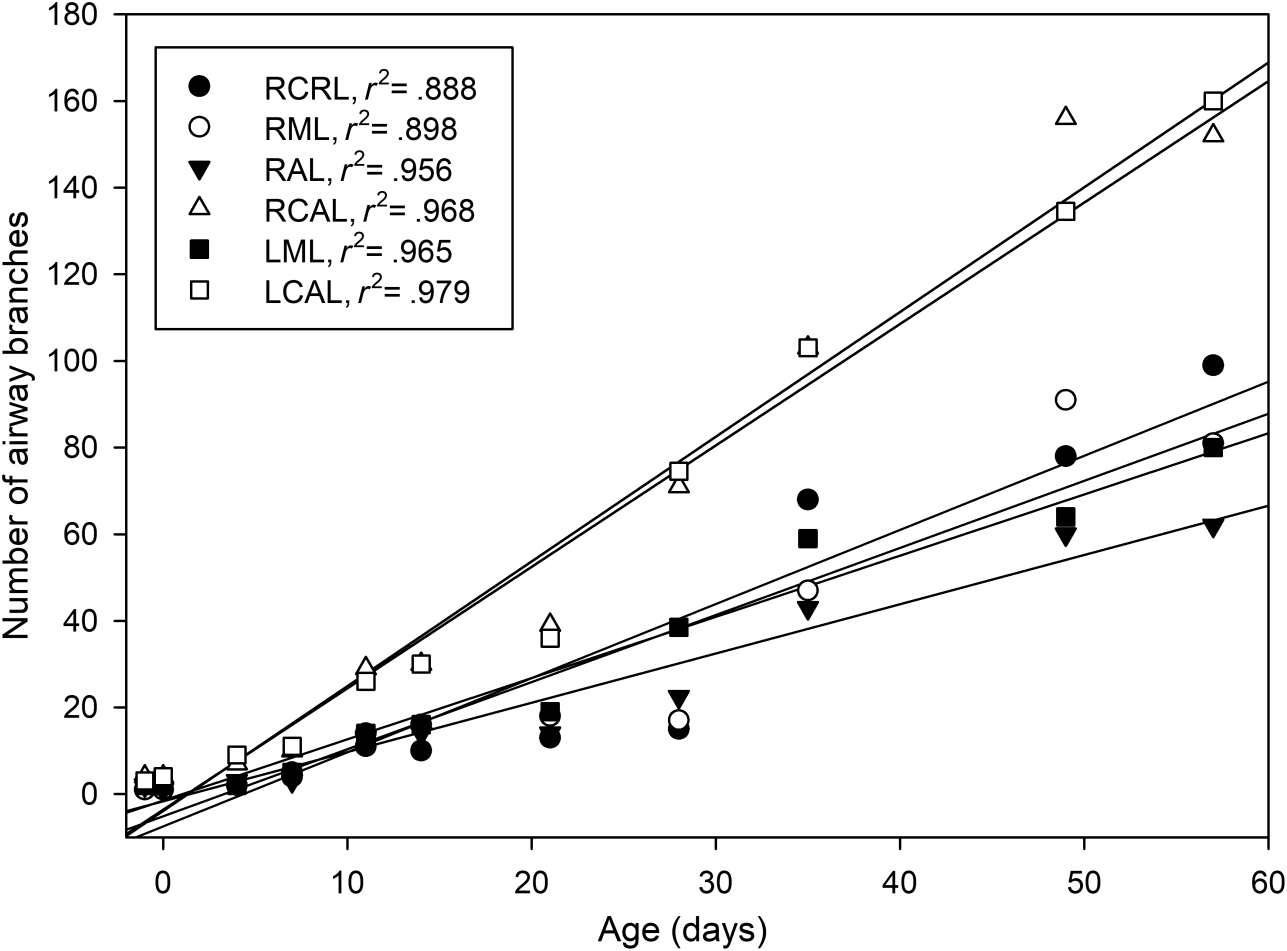
Number of airway branches for the six pulmonary lobes from 13 dpc to 57 dpn. The graphs are based on median values presented in the Table S1 and the regression lines are provided. LCAL, left caudal lobe; LML, left middle lobe; RAL, right accessory lobe; RCAL, right caudal lobe; RCRL, right cranial lobe; RML, right middle lobe.

A schema of the mature bronchial tree of *M. domestica* is presented in Figure 3. 3D reconstructions according to growth with the same scale indicate the massive size increase of the bronchial tree from 13 dpc to the adult lung (Figure 4a). Representative reconstructions of the bronchial tree of all developmental stages of *Monodelphis domestica* are shown in Figures 4, 5, 6 and details of the developing bronchial tree are given in Figures 7 and 8 and Tables 2 and 3. The generations of end‐branching airways for animals from 13 dpc to 57 dpn are presented in Figure 9 and the median, maximum, and total number of airway generations per pulmonary lobe are summarized in Table S1 and Figures 10 and S1

**TABLE 2:** Development of lobar and segmental bronchioles in the right lung.

Age (days)	No	Segmental bronchiole											
		Cranial lobe	Middle lobe	Accessory lobe	Caudal lobe								
		Eb	L1	V1	L2	L3	M1	M2	D1	D2	D3	T1	T2
13 dpc	2095e	+	+	+	+	+	+	−	+	+	−	−	+
	2095f	+	+	+	+	+	+	−	+	+	−	−	+
	2095 d	+	+	+	+	+	−	−	+	+	−	−	+
	2095 g	+	−	+	+	+	+	+	+	+	−	+	+
Neonate	1965_1	+	+	+	+	−	−	−	+	+	−	−	+
	2350_1	+	+	+	+	+	−	−	+	−	−	+	+
	2350_3	+	+	+	+	+	−	−	+	+	−	+	+
	2350_7	+	+	+	+	+	+	−	+	+	−	−	+
4 dpn	1995_5	+	+	++	+	+	+	−	+	+	−	+	+
	2257_6	+++	++	++	++	++	+	+	+	+	−	+	+
	2257_8	+	+++	++	++	+	+	+	+	+	−	+	++
7 dpn	1987_5	++	++	+++	+++	+	+	−	+	+	−	+	++
	2383_2	+++	+++	+++	+++	+	+	−	+	+	−	+	+
	2383_4	+++	+++	++	+++	++	+	+	++	+	−	++	+
11 dpn	1993_2	+++	+++	+++	++	+	+	−	+	+	−	+	++
	1993_4	+++	+++	+++	+++	+++	+	++	+++	++	+	+++	+++
	1993_3	+++	+++	+++	+++	+++	++	+++	+++	++	++	+++	+++
14 dpn	1994_8	+++	+++	+++	+++	+++	+	+	+++	++	+	+++	+++
	1994_9	+++	+++	+++	+++	+++	+	+	+++	++	+	++	+++
	1994_10	+++	+++	+++	+++	+++	++	++	+++	++	++	+++	+++
21 dpn	2040	+++	+++	+++	+++	+++	++	+	+++	++	−	+++	+++
	2037	+++	+++	+++	+++	+++	+++	++	+++	+++	+++	+++	+++
	2036	+++	+++	+++	+++	+++	+++	+++	+++	+++	++	+++	+++
28 dpn	2059	+++	+++	+++	+++	+++	++	+++	−	+++	+++	+++	+++
	2060	+++	+++	+++	+++	+++	+	++	+++	+++	+++	+++	+++
35 dpn	2065	+++	+++	+++	+++	+++	++	+++	+++	+++	+++	+++	+++
	2405	+++	+++	+++	+++	+++	+++	+++	+++	+++	+++	+++	+++
	2194	+++	+++	+++	+++	+++	+++	+++	+++	+++	+++	+++	+++
49 dpn	2049	+++	+++	+++	+++	+++	+++	+++	+++	+++	+++	+++	+++
	2402	+++	+++	+++	+++	+++	+++	+++	+++	+++	+++	+++	+++
	2403	+++	+++	+++	+++	+++	+++	+++	+++	+++	+++	+++	+++
57 dpn	2179	+++	+++	+++	+++	+++	+++	+++	+++	+++	+++	+++	+++
	2413	+++	+++	+++	+++	+++	+++	+++	+++	+++	+++	+++	+++
	2416	+++	+++	+++	+++	+++	+++	+++	+++	+++	+++	+++	+++
Adult	2095	+++	+++	+++	+++	+++	+++	+++	+++	+++	+++	+++	+++
	2117	+++	+++	+++	+++	+++	+++	+++	+++	+++	+++	+++	+++
	2419	+++	+++	+++	+++	+++	+++	+++	+++	+++	+++	+++	+++

Abbreviations: −, absent; +, existing; ++, with subsegmental bronchiole; +++, with second or more generation of subsegmental bronchiole; D, dorsal bronchiole; dpc, days post coitum; dpn, days post natum; Eb, eparterial bronchiole; L, lateral bronchiole; M, medial bronchiole; T, terminal bronchiole; V, ventral bronchiole.

**TABLE 3:** Development of lobar and segmental bronchioles in the left lung.

Age (days)	No	Segmental bronchiole												
		Middle lobe (L1)		Caudal lobe										
		Sdb	Lb	L2	L3	L4	M1	M2	M3	D1	D2	D3	T1	T2
13 dpc	2095e	+	+	+	+	−	+	−	−	+	+	−	−	+
	2095f	+	+	+	+	−	+	−	−	+	+	−	−	+
	2095d	+	+	+	+	−	+	−	−	+	+	−	+	+
	2095g	+	+	+	+	−	+	+	−	+	−	−	−	+
Neonate	1965_1	−	−	+	+	−	‐	−	−	+	−	−	−	+
	2350_1	+	+	++	+	−	−	−	−	+	−	−	−	+
	2350_3	+	+	+	+	−	+	−	−	+	−	−	+	+
	2350_7	+	+	+	+	−	−	−	−	+	−	−	−	+
4 dpn	1995_5	+	+	+	++	−	+	−	−	−	−	−	+	++
	2257_6	+	+	+	++	−	+	−	−	+	−	−	++	++
	2257_8	+	+	++	+	−	+	+	−	+	+	+	+	+
7 dpn	1987_5	+++	+	+	+	−	−	−	−	+	+	−	++	++
	2383_2	+++	+++	+++	+	−	+	+	−	+	+	−	++	++
	2383_4	+++	++	+++	+	−	+	−	−	+++	+	−	+	++
11 dpn	1993_2	+++	++	+++	+++	+	+	+−	−	+++	++	+	+	+
	1993_4	+++	+++	+++	+++	−	+	++	−	++	++	+	+++	+++
	1993_3	+++	+++	+++	+++	−	++	++	−	+++	++	+	++	+
14 dpn	1994_8	+++	+++	+++	++	++	++	++	++	+	++	−	+++	+++
	1994_9	+++	+++	+++	+++	+	++	++	−	++	++	++	+++	+++
	1994_10	+++	+++	+++	+++	+	−	+++	+	++	++	++	+++	+++
21 dpn	2040	+++	+++	+++	+++	++	+	+	+	++	++	++	+++	+++
	2037	+++	+++	+++	+++	++	+++	+++	++	++	++	+	+++	+++
	2036	+++	+++	+++	+++	++	+++	+++	++	++	++	+	+++	+++
28 dpn	2059	+++	+++	+++	+++	+	++	+++	+	+++	+++	++	+++	+++
	2060	+++	+++	+++	+++	++	+++	+++	++	+++	+++	++	+++	+++
35 dpn	2065	+++	+++	+++	+++	++	+++	+++	++	+++	+++	+++	+++	+++
	2405	+++	+++	+++	+++	++	+++	+++	++	+++	+++	+++	+++	+++
	2194	+++	+++	+++	+++	++	+++	+++	++	+++	+++	+++	+++	+++
49 dpn	2049	+++	+++	+++	+++	++	−	+++	+	+++	+++	+++	+++	+++
	2402	+++	+++	+++	+++	++	+++	+++	++	+++	+++	+++	+++	+++
	2403	+++	+++	n.e	n.e	n.e	n.e	n.e	n.e	n.e	n.e	n.e	n.e	n.e
57 dpn	2179	+++	+++	+++	+++	++	+++	+++	++	+++	+++	+++	+++	+++
	2413	+++	+++	n.e	n.e	n.e	n.e	n.e	n.e	n.e	n.e	n.e	n.e	n.e
	2416	+++	+++	n.e	n.e	n.e	n.e	n.e	n.e	n.e	n.e	n.e	n.e	n.e
Adult	2095	+++	+++	+++	+++	++	+++	+++	++	+++	+++	+++	+++	+++
	2117	+++	+++	+++	+++	++	+++	+++	++	+++	+++	+++	+++	+++
	2419	+++	+++	+++	+++	++	+++	+++	++	+++	+++	+++	+++	+++

Abbreviations: −, absent; +, existing; ++, with subsegmental bronchiole; +++, with second or more generation of subsegmental bronchiole; D, dorsal bronchiole; dpc, days post coitum; dpn, days post natum; L, lateral bronchiole; Lb, lingular bronchiole; M, medial bronchiole; n.e, not examined; Sdb, superior division bronchiole; T, terminal bronchiole.

The term “bronchus” is used to name airways with cartilage and seromucous glands in their wall (Burri, 1974). With exception of the most cranial part of the main bronchus, the airways of *M. domestica* are not supported by cartilage. Structurally, the lobar and segmental bronchi in the *M. domestica* lung equal major bronchioles. The term “bronchiole” is used to discriminate each bronchus arising from the right and left bronchi themselves. Therefore, the term bronchiole corresponds to a lobar bronchus arising from the right or left main bronchus or a segmental bronchus arising from the caudal lobe bronchi in veterinary anatomy.

#### 3.2.1 13 dpc and neonate

The lung of *Monodelphis domestica* near term at 13 dpc is at the canalicular stage of lung development. Since the lung is not inflated, the lung volume is only 0.56 mm^2^ (Table 1). The bronchial tree is not ventilated and expanded and has a low volume of 0.03 mm^3^. The developmental degree of the bronchial tree at 13 dpc (Figure 4b) and in the neonate (Figure 4c) is structurally the same. It is characterized by short lobar bronchioles branching off the main bronchi (primary/principal bronchi). The lobar bronchioles of the cranial, middle and accessory lobes and the segmental bronchioles branching off the caudal lobe bronchiole are well distinguished in the not ventilated lung of the near‐term fetus.

**FIGURE 11:**
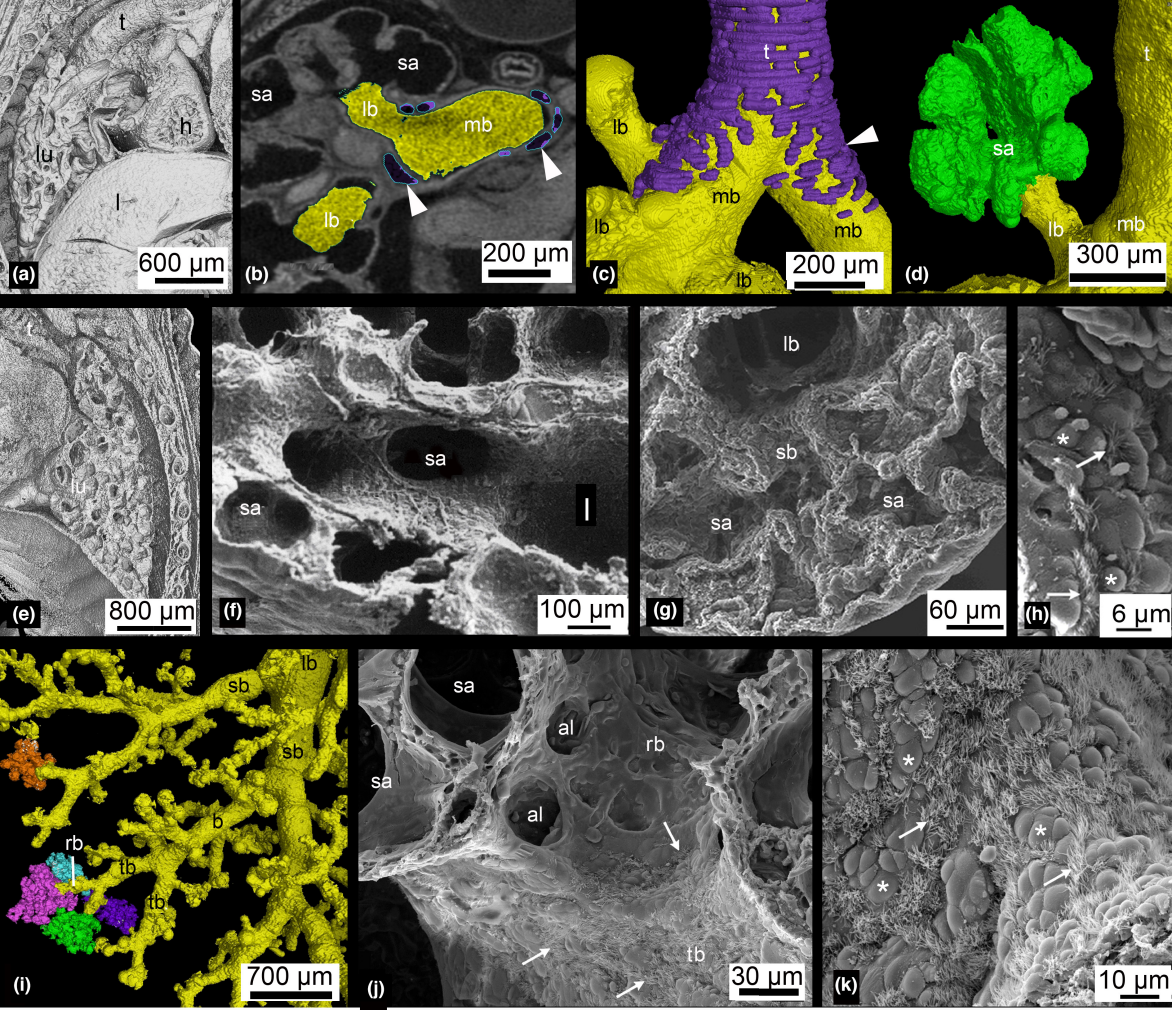
Structural development of the lung of *Monodelphis domestica* from neonate to 28 dpn. The newborn lung consists of large terminal airspaces, which branch off lobar bronchioles (a, b, d). Cartilage (indicated by filled arrow heads) is present only in the upper part of the main bronchi (b, c). At 4 dpn, the lung had entered the saccular stage of lung development, with numerous large saccules (e, f). At 7 dpn, the lung is still dominated by short branching airways opening into terminal saccules (g). First ciliated (arrow) and goblet cells (asterisk) can be found scattered in the segmental bronchioles (h). At 28 dpn, subsegmental bronchioles lead to the periphery of the lung, where they open into acini consisting of sacculi and first alveoli (i). The terminal bronchioles differentiate and first respiratory bronchioles can be identified (j). Terminal bronchioles are abundantly covered by ciliated cells (arrow) and goblet cells (asterisk). The pictures showing scanning electron images (f–h, j, k) were obtained during the authors' PhD (Szdzuy, 2006). Magnification is indicated by the scale bar. al, alveolus; h, heart; l, liver; lb, lobar bronchiole; lu, lung; mb, main bronchus; rb, respiratory bronchiole; sa, sacculus; sb, segmental bronchiole; t, trachea; tb, terminal bronchiole.

With birth, the lung of *Monodelphis domestica* becomes ventilated (Figure 11a). The volume of the air‐filled lung increases to 2.34 mm^3^ (Table 1). The volume of the expanded bronchial tree of the newborn lung is with 0.16 mm^3^ considerably larger than that of the near‐term fetus. Due to the ventilation of the lung, it is more difficult to distinguish the distal end of bronchioles from the proximal part of the terminal air spaces than in the near‐term fetus.

Bifurcation of the trachea into the right and left main bronchi marks the beginning of the bronchial tree. The main bronchi enter the hilus of the lungs where the cranial lobe bronchiole of the right lung and the middle lobe bronchiole of the left lung are branching off. The two main bronchial tubes, which have a diameter of 250–350 μm, are covered with non‐ciliated cuboidal epithelium. Near the hilum, cartilage is present (Figure 11b,c). The cartilage extends to the point where the right cranial lobe bronchiole and the left middle lobe bronchiole are branching off. Only the trachea as well as the upper two thirds of the main bronchus and the proximal part of the lobar bronchioles are solely conductive. The distal parts of the bronchioles are lined with respiratory epithelium and have conductive as well as respiratory function.

The right main bronchus divides into short cranial, middle, accessory and caudal lobe bronchioles which open directly into large terminal sacs (Figure 11b,d). The cranial lobe bronchiole originates from the dorsolateral surface of the right main bronchus. It arises above the level of the right pulmonary artery, and for this reason is named the eparterial bronchiole. The middle lobe bronchiole originates from the ventrolateral surface of the right main bronchus. The accessory lobe bronchiole originates ventromedially from the right main bronchus. It is the last branch to arise from the main bronchus at which point the right main bronchus continues caudally as caudal lobe bronchiole. Only the caudal lobe bronchiole of the right lung gives off three to five segmental bronchioles, which open directly into large terminal sacs (Figures 7 and 9a,b).

The left main bronchus divides into middle and caudal lobe bronchioles. The middle lobe bronchiole originates ventrolateral from the left main bronchus and divides into two bronchioles which supply the cranial and caudal parts of the left middle lobe. They are referred to as superior division bronchiole (Sdb) and lingular bronchiole (Lb) in accordance with the nomenclature of the human lung (Table 3). Only in one neonate specimen (1965_1) the division could not be observed. Both branches of the middle lobe bronchiole were short and opened immediately into large terminal sacs. Similar to the right lung, the caudal lobe bronchiole of the left lung continues the left main bronchus caudally and gives off three to five segmental bronchioles (Figure 9a,b). The number of branches varied from one, where the lobar bronchiole opened directly into air spaces, to four in the caudal lobes of the right and left lung, where short segmental bronchioles went off (Table S1; Figure 10).

#### 3.2.2 4 dpn

The lung has entered the saccular stage of lung development, with numerous large saccules (Figure 11e,f). The volumes of lung and bronchial tree have increased to 4.19 mm^3^ and 0.23 mm^3^. The bronchial tree of *M. domestica* at 4 dpn resembles that of the neonate, the large branching airways open after short distance into saccules (Figure 4c). However, the eparterial, middle, and accessory lobe bronchioles of the right lung give way to first short segmental bronchioles (Table 2, Figure 4c). At this point, it is partly difficult to distinguish structurally between airway and sacculus, since the newly formed segmental bronchioles are smooth‐walled and partly lined with respiratory epithelium (capillaries). The caudal lobe bronchioles of the right and left lung continue to develop. From existing segmental bronchioles, subsegmental bronchioles are formed (Figures 7 and 9c). The number of branches vary between 2 and 3 in the cranial, middle, and accessory lobes and count 7 and 9 in the right and left caudal lobes, respectively (Table S1; Figure 10).

#### 3.2.3 7 dpn

The lung is still dominated by short branching airways opening into terminal saccules (Figure 11g). However, compared to earlier developmental stages, a more differentiated bronchial tree is present (Figure 4d). The main bronchi are lined with one‐layered cuboidal epithelium. Only in the proximal part, cartilage is present. The distal parts the lobar bronchioles are lined with respiratory epithelium. Bronchioles in proximity to saccules have an extensive capillary bed subjacent to the epithelium.

The lung volume increases with 5.63 mm^3^ moderately, but the volume of the bronchial tree (0.62 mm^3^) more than doubles compared to 4 dpn. In all lung lobes, segmental bronchioles are formed, giving rise to several subsegmental bronchioles (Figures 7 and 9d; Tables 2 and 3). The number of branches increases throughout the lung lobes, varying from 3 to 5 in cranial, middle, and accessory lobes and 10 to 11 in the right and left caudal lobes (Figure 10). Ultrastructural examination reveals that epithelium typical for the terminal airways is developing. First ciliated and goblet cells can be found scattered in the segmental bronchioles (Figure 11h).

#### 3.2.4 11 dpn, 14 dpn, and 21 dpn

**FIGURE 12:**
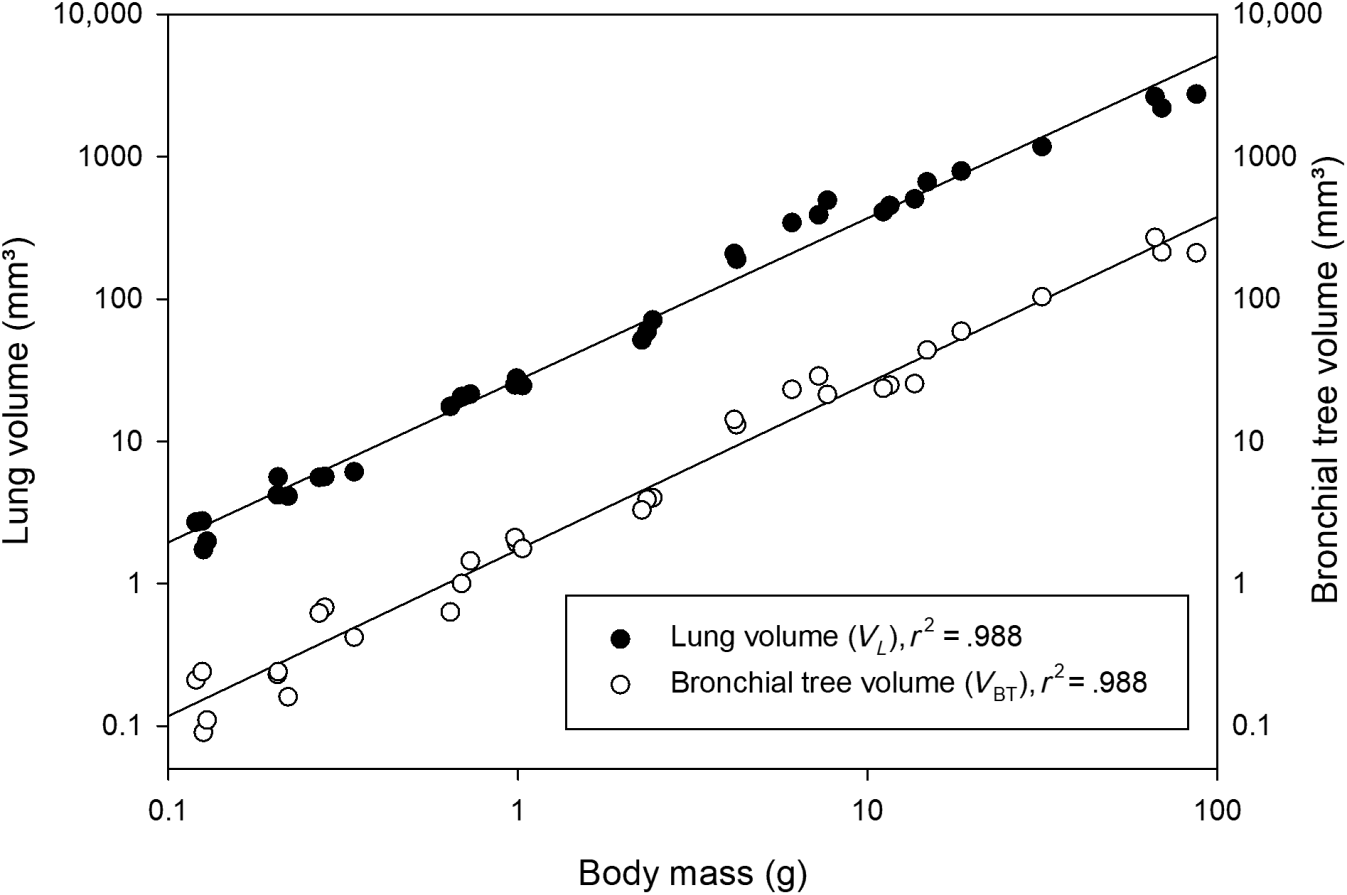
Double logarithmic plots of the lung volume (*V*_L_) and bronchial tree volume (*V*_BT_) against body mass for *Monodelphis domestica* in the postnatal period. The graphs are based on individual animal data and the regression lines are provided.

In this developmental period, the lung volume is increasing steadily from 20.58 mm^3^ at 11 dpn to 58.84 mm^3^ at 21 dpn. Simultaneously, the volume of the bronchial tree increases continuously from 1.00 to 3.90 mm^3^ (Table 1; Figure 12).

From 11 dpn to 21 dpn, the lung is still at the saccular stage of lung development, however, increases markedly in complexity. This increase in complexity is concurrent with an increase in saccular number and decrease in the size of the saccules. The bronchial tree is characterized by enhanced architectural complexity (Figure 5a–c). Segmental bronchioles, often with first, second, or more generations of subsegmental bronchioles, are present in all pulmonary lobes (Figure 9e–g; Tables 2 and 3). By 14 and 21 days, the main bronchi measure 250–300 μm in diameter and the bronchiolar epithelium resembles that of the newborn lung. Cartilage supports the main bronchi only to the first branch arising from the main bronchus. The surrounding stroma contains smooth muscle cells. The lobar bronchioles branching off from the proximal part of the main bronchus have solely conductive function. After a renewed dichotomy, in segmental bronchioles, the bronchiolar epithelium is partially lined with respiratory epithelium. From the segmental bronchioles, short terminal subsegmental bronchioles branch off and open immediately into the saccules. These terminal bronchioles are characterized by one‐layered cuboidal epithelium interspersed with capillaries and a thin circular layer of smooth muscle cells underlying the bronchiolar epithelium. The cuboidal epithelium of the distal part of the terminal bronchioles is densely ciliated.

The higher complexity of the lung, especially at 14 and 21 dpn, is mirrored by a further increase in median, maximum generation, and the number of branches in all lung lobes (Table S1; Figure 10). At 21 dpn, the number of branches is varying from 13 to 19 in cranial, middle, and accessory lobes and 39 and 36 in the right and left caudal lobes, respectively.

#### 3.2.5 28 dpn

Between 28 and 35 dpn, substantial changes take place in the lung of *Monodelphis domestica*. With the start of alveolarization at 28 dpn, the lung is at the transition from the saccular to the alveolar stage of lung development. Besides the predominant saccules, first alveoli can be identified at 28 dpn and even first respiratory bronchioles are apparent (Figure 11j). At 28 dpn, the lung volume (199.19 mm^3^) has tripled compared to 21 dpn and also the volume of the bronchial tree has increased considerably to 13.67 mm^3^. The complexity of the bronchial tree is increasing further (Figures 5d and 11i). The well‐developed bronchial tree with 12 generations of bronchioles extends to the periphery of the lung (Figure 9h, Tables 2 and 3). The number of branches increases furthermore in all lung lobes, varying between 15 and 38 in the cranial, middle, and accessory lobes and almost doubling with 71 and 74.5 in the right and left caudal lobes, respectively (Table S1; Figure 10).

The main bronchi have a diameter of 250 μm and are lined with one‐layered cuboidal epithelium. A layer of smooth muscle cells is situated beneath the mucosa. For stability, cartilage surrounds the layer of muscle cells, but only in the proximal part of the main bronchus up to the point where the first lobar bronchioles branch off. The lobar bronchioles have a diameter of 100–125 μm and consist of one‐layered cuboidal epithelium and a circular layer of muscle cells. Segmental bronchioles are similar in structure, however, smaller with 60–70 μm in diameter. The most distal airways are the terminal bronchioles. They have a diameter of 40–45 μm and are lined with one‐layered cuboidal epithelium and only few muscle cells underlie the epithelium. Ciliated epithelium consisting of ciliated cells and goblet cells, typical for the terminal bronchioles in mammals, can be seen abundantly at 28 dpn (Figure 11j,k).

#### 3.2.6 35 dpn

At 35 dpn, the lung has fully reached the alveolar stage of lung development; no saccules are present anymore. The volumes of the lung (388.59 mm^3^) and of the bronchial tree (23.16 mm^3^) increase steadily and nearly double compared to 28 dpn (Figure 12). The structural development of the bronchial tree seems to be completed. All segmental bronchioles and subsegmental bronchioles in all lung lobes are present (Figure 6a; Tables 2 and 3). The complex bronchial tree extends with up to 13 generations to the lung periphery (Figure 9i). The number of branches increases distinctly in all lung lobes, varying between 43 and 68 in the cranial, middle, and accessory lobes and 103 in the caudal lobes of the right and left lung (Table S1; Figure 10).

**FIGURE 13:**
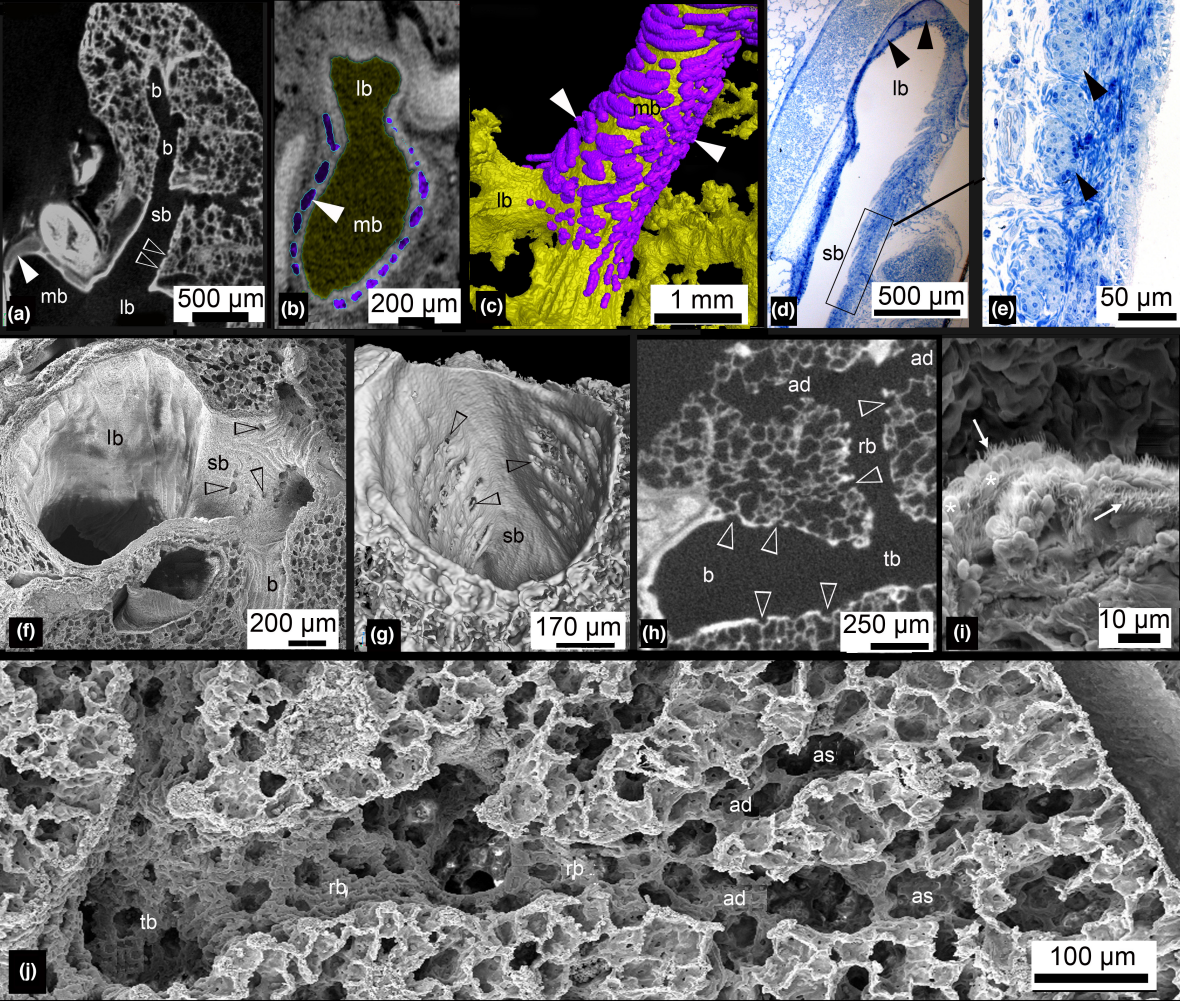
Structural development of the lung of *Monodelphis domestica* from 35 dpn to adult. At 35 dpn, a complex bronchial tree with several generations of subsegmental bronchioles is present. In the walls of segmental bronchioles, alveoli (open arrow heads) can be seen (a). Cartilage (filled arrow heads) supports the main bronchus up to the point where the cranial lobe bronchus is branching off (b, c), but a small amount of cartilage is present in the proximal part of lobar and segmental bronchioles as well (d, e). At 57 dpn and in adults, alveoli can be found in major bronchioles with considerably thick bronchiolar epithelium (h) and even in the walls of large segmental bronchioles (f, g). The terminal bronchioles are densely lined with ciliated epithelium (arrow) and goblet cells (asterisk) (i). Typical terminal airways, consisting of terminal bronchioles branching into respiratory bronchioles, followed by alveolar ducts and finally opening to alveolar sacs can be seen in the adult lung (j). The pictures showing scanning electron images (f, i, and j) were obtained during the authors' PhD (Szdzuy, 2006). Magnification is indicated by the scale bar. ad, alveolar duct; as, alveolar sac; b, bronchiole; lb, lobar bronchiole; mb, main bronchus; rb, respiratory bronchiole; sb, segmental bronchiole; tb, terminal bronchiole.

**FIGURE 14:**
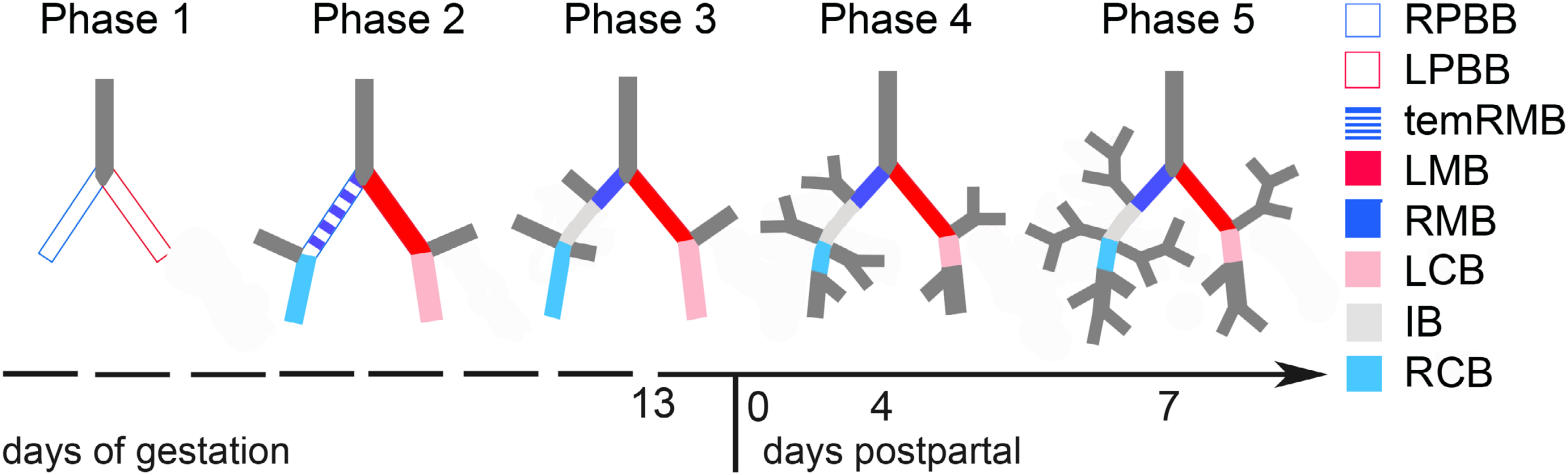
Five phases of lung development based on the bronchial morphology. In phase 1, two primary bronchial buds are formed. In phase 2, two lobar bronchioles are on each side. All lobar bronchioles are completed in phase 3. Segmental bronchioles are present in phase 4. Phase 5 has any of the subsegmental bronchioles. The newborn *Monodelphis domestica* has already finished phase 3 and enters phase 4 at 4 dpn. Subsegmental bronchioles (phase5) are present at 7 dpn. IB, intermediate bronchus; LCB, left caudal bronchiole; LMB, left main bronchus; LPBB, left primary bronchial bud; RCB, right caudal bronchiole; RMB, right main bronchus; RPBB, right primary bronchial bud; temRMB, temporary RMB, branch from the tracheal bifurcation to the base of the right middle lobe. Modified from Fujii et al. (2020).

A reconstruction of cartilage in the bronchial tree at 35 dpn, reveals no significant differences to the situation described in the newborn lung. Cartilage supports the main bronchus up to the point where the cranial lobe bronchus is branching off (Figure 13b,c). It is irregularly arranged in crescent‐shaped plates and islands. Although under the limited resolution of the μCT, no cartilage was evident beyond this point, histological investigation indicated that a small amount of cartilage is present in the proximal part of lobar and segmental bronchioles as well (Figure 13d,e).

With progressing alveolarization, a unique characteristic of the lung structure of *M. domestica* becomes visible. In the walls of segmental bronchioles, alveoli can be seen (Figure 13a).

#### 3.2.7 49 and 57 dpn

The lung volumes at 49 and 57 dpn are increasing furthermore to 452.66 and 790.89 mm^3^, so are the volumes of the bronchial tree (24.78 cm^3^ and 59.28 mm^3^).

Structurally, no major changes to 35 dpn can be observed (Figure 6b–d). The mature bronchial tree is still extending by forming new bronchioles, meeting the requirements of the growing lung. Up to 18 generations of bronchioles extend to the lung periphery of the caudal lobes at 49 and 57 dpn (Figure 9j,k). The number of branches increases in all lung lobes as well, varying between 62 and 99 in the cranial, middle, and accessory lobes and 152 and 160 in the right and left caudal lobes at 57 dpn (Table S1; Figure 10).

By 57 dpn, the main bronchi have doubled in diameter to 500 μm. They are lined with one‐layered cuboidal, partly columnar epithelium. A thick layer of smooth muscle cells, separated by connective tissue, underlies the bronchiolar epithelium. Embedded in connective tissue seromucous glands can be found. The proximal part of the main bronchi is supported by cartilage. Cartilage is present up to the point where the middle lobe bronchioles branch off in the right and left lung, respectively. Compared to earlier stages, the cartilage has extended from the first to the second branch of the right main bronchus. The lobar and segmental bronchioles have a diameter of 300–400 μm. They are lined with cuboidal epithelium and smooth muscle cells underlie the epithelium. The terminal bronchioles have a diameter of 50–100 μm and only a thin layer of smooth muscle cells can be found. With the progressive formation of alveoli, numerous respiratory bronchioles develop. They have a diameter of 50–100 μm. Mature acini, consisting of alveolar ducts opening to alveolar sacs were easily distinguished by day 49 and 57 dpn and appeared to be no different from those observed in the lung of adult animals (Figure 13j). The terminal bronchioles were densely lined with ciliated epithelium, typical for the bronchiolar epithelium of the mature mammalian lung (Figure 13i).

#### 3.2.8 Adult

Finally, the adult lung has a lung volume of 2631.63 mm^3^ and a bronchial tree volume of 213.35 mm^3^ (Table 1; Figure 12).

The bronchial tree of the adult *Monodelphis domestica* is similar in structure to the bronchial tree at 57 dpn. However, the lumina of the bronchi increase in size and the bronchial walls are getting thicker. The main bronchi measure 1.5 mm in diameter. The bronchial epithelium is columnar. In the proximal part, circular layers of smooth muscle cells and cartilage are underlying the bronchial epithelium. The bronchial musculature causes extensive infoldings. The lobar bronchioles, branching off from the main bronchus, measure 600 μm in diameter. Their walls are lined with cuboidal epithelium and consist of a thick layer of smooth muscle cells comparable to those of the main bronchi. The segmental bronchioles measure 200–400 μm in diameter and the bronchiolar epithelium is supported by a circular layer of smooth muscle cells, which is thinner than that of the main bronchi or lobar bronchioles.

In the mature lung, the peculiarity of alveoli located in the walls of larger airways becomes even more evident. Shortly after branching off from the lobar bronchioles, the walls of segmental bronchioles are perforated by numerous alveoli (Figure 13f). These irregular formations of alveoli are common in the adult lung. Alveoli can be found in terminal bronchioles, major bronchioles with considerably thick bronchiolar epithelium (Figure 13h) and even in the walls of large segmental bronchioles (Figure 13g).

With the maturation of the lung, the musculature expands to the proximal part of the bronchial tree. Even in terminal bronchioles, a thin layer of smooth muscle cells underlies the bronchiolar epithelium. The terminal bronchioles are lined with cuboidal epithelium which consists abundantly ciliated cells (Figure 13i). Terminal and respiratory bronchioles are the most distally located airways and measure 200 μm in diameter. The respiratory bronchioles have regular alveoli at their sides. From the respiratory bronchioles, smaller alveolar ducts (100 μm) branch off. They open into alveolar sacs, from which alveoli radiate in a concentric way (Figure 13j).

## 4 DISCUSSION

It is generally accepted that viability of the newborn mammal depends on an adequately developed respiratory apparatus (Frappell & MacFarlane, 2006; Simpson et al., 2013). The lung in the newborn Gray short‐tailed opossum is at the canalicular stage of lung development and structurally immature. However, the lung is functioning and takes over the main part of respiration, supported by cutaneous respiration. The exact amount of cutaneous gas exchange is unknown for *Monodelphis domestica*, but it can be assumed that it is between 30% reported for the tammar wallaby and 90%–100% reported for the fat‐tailed dunnart (MacFarlane & Frappell, 2001; Simpson et al., 2011).

According to the low developmental degree of the lung, also other organ systems (heart, kidney, liver, skin, and skeleton) in the newborn Gray short‐tailed opossum have a low degree of differentiation (see Ferner et al., 2017).

The lung of the newborn Gray short‐tailed opossum is characterized by a low lung volume. Similar low lung volumes were reported for other marsupial neonates (Makanya et al., 2003, 2007; Runciman et al., 1998; Simpson et al., 2013).

The volumes of the lung and of the bronchial tree increased steadily during the postnatal period (Figure 12). *Monodelphis domestica* demonstrates a 550‐fold increase in body weight, but the lung volume increases ~1100‐fold through development. In larger marsupial species, an even greater increase in lung volume was reported, 3800‐fold in the tammar wallaby (Runciman et al., 1998) and 8000‐fold in the quokka (Makanya et al., 2003).

The bronchial tree of *Monodelphis domestica* corresponds to the six lung lobes. The lobation of the lung is similar to previous descriptions of many marsupial species and the monotreme short‐beaked echidna (Perry et al., 2000). Cope et al. (2001) states that a right lung divided into four lobes is a common pattern found in most of the smaller marsupial species. Larger marsupials such as kangaroos, wallabies, and koala have fewer lobes in the right lung (Table 4). The bronchial pattern of *Monodelphis domestica* indicates that the left lung consists of two lobes, even when no interlobular fissure is present. The first lobar bronchiole of the left lung is considered to be a hyparterial bronchiole, in contrast to the eparterial bronchiole of the cranial lobe of the right lung, and originates at the same level as that of the right middle lobe bronchiole. Due to the common features, the bronchiole arising from the most cranial portion of the left main bronchus can be considered as left middle lobe bronchiole.

**TABLE 4:** Lobation in the right and left lungs of various mammals.

Family/genus/species	Lobes of the right lung				Lobes of the left lung				References
	Cr	M	A	Ca	Cr	M	A	Ca	
Monotremata									
*Ornithorhynchus anatinus*	+	+	+	+	+	+	−	+	Ferner et al. (2009)
*Tachyglossus aculeatus*	+	+	+	+	−	+	−	+	Perry et al. (2000)
Marsupialia									
*Marmosa elegans*	+	+	+	+	−	+	−	+	Sonntag (1921a)
*Philander opossum*	+	+	+	+	−	+	−	+	Sonntag (1921a)
*Caenolestes obscurus*	+	+	+	+	−	−	−	+	Osgood (1921)
*Dasyurus viverrinus*	+	+	+	+	−	+	−	+	Owen (1868)
*Dasycercus cristicauda*	+	+	+	+	−	+	−	+	Owen (1868)
*Perameles nasuta*	+	+	+	+	−	−	−	+	Owen (1868)
*Choeropus castanotis*	+	+	+	+	−	−	−	+	Parsons (1903)
*Petaurista*	+	+	+	+	−	+	−	+	Owen (1868)
*Potorous*	+	+	+	+	−	+	−	+	Owen (1868)
*Trichosurus vulpecula*	+	+	+	+	−	+	−	+	Cooke and Alley (2002)
*Macropus eugenii*	+	+	+	+	−	+	−	+	Szdzuy (2006)
*Macropus giganteus*	+	−	+	+	−	+	−	+	Sonntag (1921a)
*Macropus bennetti*	+	−	+	+	−	+	−	+	Sonntag (1921a)
*Dendrolagus*	+	−	+	+	−	+	−	+	Sonntag (1921a)
*Didelphis marsupialis*	+	+	+	+	−	+	−	+	Bremer (1904), Pantoja et al. (2020)
*Vombatus ursinus*	+	−	−	+	−	−	−	+	Owen (1868)
*Phascolarctos cinereus*	+	+	−	+	−	+	−	+	Forbes (1881), Sonntag (1921b)
Placentalia									
*Mus musculus*	+	+	+	+	−	+	−	+	Vasilescu et al. (2012)
*Rattus norwegicus*	+	+	+	+	−	+	−	+	Counter et al. (2013)
*Mesocricetus auratus*	+	+	+	+	−	+	−	+	Szdzuy (2006)
*Cavia aperea*	+	+	+	+	−	+	+	+	Szdzuy (2006)
*Galea musteloides*	+	+	+	+	+	+	+	+	Wallau et al. (2000)
*Castor fiber*	+	+	+	+	−	−	−	+	Wallau et al. (2000)
*Myocastor coypus*	+	+	+	+	+	+	+	+	Wallau et al. (2000)
*Octodon degu*	+	+	+	+	+	+	−	+	Wallau et al. (2000)
*Ctenodactylus gundi*	+	+	+	+	+	+	−	+	Wallau et al. (2000)
*Suncus murinus*	+^1^	+	+	+	−	−	−	+	Szdzuy (2006)
*Tupaia belangeri*	+	+	+	+	+	+	−	+	Ferner et al. (2010)
*Macroscelides proboscideus*	+	+	+	+	+	+	−	+	Szdzuy (2006)
Leporidae	+	+	+	+	−	+	−	+	Simons (1996)
*Felis cattus*	+	+	+	+	−	+	−	+	Caccamo et al. (2007)
*Canis familiaris*	+	+	+	+	−	+	−	+	Ishaq (1980)
*Sus scrofa*	+^1^	+	+	+	−	+	−	+	Nakakuki (1994a)
*Ovis aries*	+^1^	−	−	+	−	+	−	+	Tawhai et al. (2004)
*Bos taurus*	+^1^	+	+	+	−	+	−	+	Nakakuki (1994b)
*Equus caballus*	+	+	+	+	+	+	+	+	Nakakuki (1993a)
*Cervus nippon*	+^1^	+	+	+	+	+	−	+	Nakakuki (1993b)
*Phoca hispida*	+	+	+	+	+	+	−	+	Smodlaka et al. (2009)
*Callithrix jacchus*	+	+	+	+	−	+	−	+	Falcão et al. (2018)
*Macaca fuscata*	+	+	+	+	−	+	−	+	Nakakuki (1986)
*Gorilla gorilla*	+	+	+	+	−	+	−	+	Nakakuki (1991)
*Pan troglodytes*	+	+	−	+	−	+	−	+	Nakakuki (1992)
*Homo sapiens*	+	+	−	+	−	+	−	+	Tawhai et al. (2004)

Abbreviations: A, accessory; Ca, caudal; Cr, cranial; M, middle.

Lobation of the left lung in marsupials is described in many studies as one or two lobes (Table 4). These descriptions are apparently based on the external appearance of the lung as branching of the bronchial tree is not mentioned. However, even if the studies describe only one lobe, an examination of the bronchial tree would likely show that it actually consists of two fused lobes (Cope et al., 2001) as proven for *M. domestica* in this study.

In placental mammals, there is more variability with respect to bronchial tree architecture and lobation of the lung (see Table 4). Nakakuki (1975, 1980) examined many mammalian lungs in order to establish the fundamental structures of their bronchial ramifications. The most common and probably plesiomorphic bronchial pattern in mammals consists of cranial, middle, ventral (accessory), and caudal lobe bronchioles in the right lung and a well‐developed middle lobe bronchiole (cranial lobe is often synonymous with the middle lobe) and caudal lobe bronchioles in the left lung (Nakakuki, 1975). There are some variations from this bronchial system in placental mammals, such as tracheal bronchioles, subdivision of pulmonary lobes and the presence of accessory and cranial lobes in the left lung (Nakakuki, 1980, 1993a; Szdzuy, 2006; Wallau et al., 2000).

Even if *M. domestica* represents a basal mammalian species, its bronchial tree bears some similarities to the bronchial tree of the human lung (see Fujii et al., 2020; Netter & Mühlbauer, 2011). In the right lung, the cranial, middle, caudal, and accessory lobe correspond to the superior, middle, inferior lobe, and the right medial basal segment (B^7^) of the human lung. In the left lung, the middle and caudal lobes of the *M. domestica* lung correspond to the superior and inferior lobes in humans, including the subdivision of the superior lobe bronchus into superior division bronchus (Sdb) and lingular bronchus (Lb).

This is the first study providing three‐dimensional reconstructions of the bronchial tree of the developing lung in a marsupial species. So far, the only available descriptions concerning bronchial development in marsupials focused on the histology and ultrastructure of these structures (Cooke & Alley, 2002; Krause & Leeson, 1973, 1975). The structural descriptions of bronchioles range from non‐ciliated columnar epithelium in the newborn American opossum (*Didelphis virginiana*) to highly vascularized and covered by ciliated and non‐ciliated epithelial cells in the newborn possum (*Trichosurus vulpecula*). Our observations on the bronchial epithelium of the newborn Gray short‐tailed opossum generally confirm these findings. However, ciliated cells were not present in the newborn *M. domestica* lung. First scattered patches of ciliated cells were found around 7 dpn in *M. domestica*, similarly to the observations of Krause and Leeson (1973, 1975), who reported ciliated cells by 9 days in the American opossum. In most placental species, ciliated cells are usually present at birth or shortly after in the conducting portion of the lung (Castleman & Lay, 1990; Kahwa et al., 2000; Sorokin, 1968; Szdzuy, 2006; Winkler & Cheville, 1984). Starting with the alveolar period (28 dpn) until adulthood, the bronchiolar walls of the *M. domestica* lung were densely lined with ciliated epithelium, similar to that found in the adult lungs of monotreme (Perry et al., 2000), marsupial (Cooke & Alley, 2002; Krause & Leeson, 1975), and placental species (Iovannitti et al., 1985; Jeffery, 1998; Tyler & Plopper, 1985; Wright et al., 1983).

Cartilage was found only in the upper part of the main bronchi in the lung of *M. domestica*. Similarly, Hughes and Hall (1984) mentioned that, at birth, cartilage rings had formed in the primary bronchi of the lung of the brushtail possum. However, Cooke and Alley (2002) could not observe cartilage until 57 days in the same species.

Information about the structural modifications during bronchial tree development in marsupials are sparse. Bronchioles could be clearly defined in the lung of a possum by 63 days and secondary bronchi were observed by 15 days in the American opossum and by 105 days in the Brushtail possum. These secondary bronchi branched into respiratory bronchioles followed by alveolar ducts similar to those observed in the lung of adult animals (Krause & Leeson, 1973, 1975). Thus, the development of the bronchi of the American opossum and the Brushtail possum has similarities to that observed in the Gray short‐tailed opossum. However, in the latter species, the development of the bronchial tree appears to be faster. A mature bronchial tree with respiratory bronchioles and alveolar ducts is present in *M. domestica* already by 35 dpn.

Presumably because of its short gestation period (13.5 days), the lungs of the newborn Gray short‐tailed opossum consist only of the conducting portion of the bronchial tree which is modified for respiratory exchange. During the first 35 days of development, differentiation and expansion of this modified bronchial tree takes place. Air chambers immediately adjacent to established bronchioles differentiate further and become incorporated into the bronchial tree and new air chambers develop at the most distal end of the bronchial system. Krause and Leeson (1975) state that type I and type II pneumocytes, which make up the walls of the air chambers, appear to be specialized for respiratory function, but they also may retain the potential to divide. High mitotic activity in the cuboidal type II pneumocytes suggests that they might provide new lining cells for adjacent newly formed air chambers (Krause & Leeson, 1975). In placental mammals, similar areas of cuboidal cells and the epithelium of terminal and respiratory bronchioles have been described forming lining epithelium of alveoli (Boyden & Tompsett, 1961; Sorokin, 1968). Thus, the newly formed bronchiolar lining epithelium of *M. domestica* may be derived from adjacent portions of the bronchial tree or could be derived from a dedifferentiation of previous respiratory epithelium. The peculiarity of alveoli located in the walls of large conducting airways, found in the mature lung of *M. domestica*, might be a remnant of the transformation of former respiratory epithelium into bronchiolar epithelium. This special feature of the marsupial lung most likely results from the postnatal formation of the bronchial tree within a functioning lung.

In placental mammals, the development of the bronchial tree is completed in utero. In humans, the preacinar airway branching pattern is completed by about the 17th week of intrauterine life (Jeffery, 1998). Fujii et al. (2020) studied the morphogenesis of the bronchial tree in humans until the end of the embryonic period. They proposed five developmental phases for the bronchial tree according to the degree of bronchus formation, which we adopted for the bronchial development in *M. domestica* (see Figure 14). In phase 1, the primary bronchus has no lobar swellings, it forms an almost symmetric Y‐shape. In phase 2, the primary bronchus has lobar swellings that emerge from the middle of each bud, still exhibiting almost total symmetry. In phase 3, the bronchus has all six distinct lobar swellings, indicating the future lobar bronchioles. At this time, the right and left primary bronchi show characteristic asymmetry. In phase 4, the segmental bronchioles begin to emerge from the lobar bronchioles, without any subsegmental bronchioles. In phase 5, finally the subsegmental bronchioles appear.

Applying this system to the Gray short‐tailed opossum, the near‐term (13 dpc) and newborn bronchial tree can be assigned to Phase 3. At this time, all lobar bronchioles and even first segmental bronchioles branching off the caudal lobes can be recognized, indicating that phase 3 is nearly completed. In the 4‐days‐old *M. domestica* several segmental bronchioles branch off the lobar bronchioles, assigning the bronchial tree to phase 4 of this developmental system. Within 7 days, subsegmental bronchi can be found in all lung lobes, clearly indicating that phase 5 has been attained.

Whereas the fundamental structure of the proximal part of the bronchial tree, consisting of the lobar and segmental bronchi, is determined at the embryonic period in humans (Fujii et al., 2020), *M. domestica goes* through this developmental stage during the first postnatal weeks. By 14 days, a bronchial tree consisting of lobar, segmental, and subsegmental bronchioles has been established. At this time, the young Gray short‐tailed opossums, which were firmly attached to the maternal teat before, first detach from the mother. With 35 days, the bronchial tree can be considered to be mature and possesses all characteristics of an adult lung. This developmental time coincides with the presence of pelage and first leaving of the nest.

The subsequent development of the bronchial tree into the final mature bronchial tree in *M. domestica* seems to follow similar mechanisms as described for the bronchial development in the mouse (Metzger et al., 2008) or in humans (Warburton, 2021). The bronchial tree develops by branching of the airway epithelium into surrounding mesenchyme. Branching morphogenesis of the upper airway as well as saccular formation and later alveolarization in the lower airway seem to rely on the same fundamental process: epithelial extrusion through an orifice. The orientation and relative stiffness of the orifice boundary determines the stereotype of upper airway branching (Warburton, 2021). Metzger et al. (2008) described three local modes of branching in three different sequences which are used in the developing lung. These branching modes (domain branching, planar bifurcation, and orthogonal bifurcation) create a different arrangement of branches and serve specific functions in lung design. The processes described as domain branching in the mouse between embryonic day 11 and 15 are remarkably similar to the branching pattern seen in *M. domestica* during the first 14 postnatal days. The daughter branches (segmental bronchioles) form in rows (“domains”) at different positions around the circumference of the primary bronchus. The morphogenesis of the bronchial tree of *M. domestica* has already started in the prenatal period (see Figure 14). In the left lung, the first secondary bronchiole (L1) has branched off the lateral aspect of the primary bronchus. Additional branches sprouting distal to L1 can be distinguished at birth or in the following days, creating a row of lateral secondary branches numbered in the proximal–distal sequence in which they form (L2, L3, and L4). The same applies for another row, which forms along the dorsal surface of the primary bronchus (D1, D2, and D3). A third row begins to sprout from the medial surface of the primary bronchus, forming the medial bronchiole system (M1, M2, and M3). Although many tertiary branches also form by domain branching, as seen in the middle and accessory lobes of the right lung in *M. domestica*, many tertiary or later generation branches form by a different mode in which the tip expands and bifurcates along the anterior–posterior axis. These pair of tertiary branches bifurcate again either again in the same axis (planar bifurcation) or in a ~ 90° rotated plane (orthogonal bifurcation) forming four quaternary branches. Beside bifurcation, also trifurcation of the branches could be observed.

The lung volumes reported in this study might not correspond exactly to the functional lung volumes of living animals. Potential limitations with respect to the method of fixation need to be discussed. In smaller developmental stages (0–28 dpn), the specimens were totally immersed in fixative after severing the head to allow fixative inflow. In these animals, the lung volume might be lower than the functional value due to the lungs deflating below functional residual capacity (FRC) at death (Simpson et al., 2013). Since the inflation degree of the lung cannot be controlled with that method, a higher variation in lung volumes might result. In older stages, the lungs were fixed by installation via the trachea with a tracheal pressure of 20 cm H_2_O. This may lead to an overestimation of lung volume since lungs inflated with liquid have a larger compliance and are easier to distend than air‐filled lungs (West, 1995). Regardless of the preservation methods, the lung volumes calculated for *Monodelphis domestica* are comparable to lung volumes reported for other marsupial pouch young of similar size.

## 5 CONCLUSION

The asymmetry of the right (four lobes) and the left lung (two lobes), as present in *Monodelphis domestica*, is common in other basal taxa and many distantly related mammalian groups (Table 4). Therefore, the bronchial tree of *M. domestica* could be considered as plesiomorphic for Mammalia. In marsupials, the process of branching morphogenesis, which takes place intrauterine in the placental fetus, is shifted to the postnatal period and therefore more easily accessible for investigation. The development of the bronchial tree from an embryonic developmental degree to the final adult lung structure, takes place during the functional state in a continuous morphogenetic process. Similarities to the branching morphogenesis described in mouse and humans suggest that this process is highly conservative within mammalian evolution and controlled by a genetically encoded subroutine. Lung maturation in general and the branching morphogenesis in particular in placental and marsupial mammals follow a similar pattern and both animal groups are at a similar stage of lung development when the young first gain independence.

## AUTHOR CONTRIBUTIONS

Kirsten Ferner: design, dissection of lungs, 3D reconstructions, data collection and analysis/interpretation, article writing, and editing; Kristin Mahlow: staining, sample preparation and acquisition of μCT scans, and critical review of the article.

## CONFLICT OF INTEREST STATEMENT

The authors declare no conflict of interest.

### OPEN RESEARCH BADGES

This article has earned an Open Data badge for making publicly available the digitally‐shareable data necessary to reproduce the reported results. The data is available at https://doi.org/10.7479/0yk5‐wr21.

## Supporting information


Figure S1

Table S1

## Data Availability

The data that support the findings of this study, additional images, and videos of 3D reconstructions of bronchial trees are available under https://doi.org/10.7479/0yk5‐wr21.
