# Supplementary material for: 3D reconstruction of the bronchial tree of the Gray short‐tailed opossum (Monodelphis domestica) in the postnatal period
Source: J Anat. 2023 Jul 27;243(6):910–35. doi: 10.1111/joa.13928 (PMC10641052; doi:10.1111/joa.13928)
Supplement: Supplementary file 2 — Table S1 [file JOA-243-910-s002.docx]

**Supplementary data**
